# *De Novo* Assembly and Genome Analyses of the Marine-Derived *Scopulariopsis brevicaulis* Strain LF580 Unravels Life-Style Traits and Anticancerous Scopularide Biosynthetic Gene Cluster

**DOI:** 10.1371/journal.pone.0140398

**Published:** 2015-10-27

**Authors:** Abhishek Kumar, Bernard Henrissat, Mikko Arvas, Muhammad Fahad Syed, Nils Thieme, J. Philipp Benz, Jens Laurids Sørensen, Eric Record, Stefanie Pöggeler, Frank Kempken

**Affiliations:** 1 Department of Genetics & Molecular Biology in Botany, Institute of Botany, Christian-Albrechts-University at Kiel, Kiel, Germany; 2 Architecture et Fonction des Macromolécules Biologiques, Aix-Marseille Université, 13288 Marseille, France; 3 Centre National de la Recherche Scientifique, CNRS UMR 7257, 13288 Marseille, France; 4 VTT Technical Research Centre of Finland Ltd, Tietotie 2, FI-02044 VTT, Espoo, Finland; 5 Biocomputing Platforms Ltd, Tekniikantie 14, FI-02150, Espoo, Finland; 6 Holzforschung München, TUM School of Life Sciences Weihenstephan, Technische Universität München, Hans-Carl-von-Carlowitz-Platz 2, Freising, Germany; 7 Department of Chemistry and Bioscience, Aalborg University, Niels Bohrs Vej 8, DK-6700 Esbjerg, Denmark; 8 INRA, UMR1163 Biotechnologie des Champignons Filamenteux, Aix-Marseille Université, Polytech Marseille, 163 avenue de Luminy, CP 925, 13288 Marseille Cedex 09, France; 9 Aix-Marseille Université, INRA, UMR1163 Biotechnologie des Champignons Filamenteux, Faculté des Sciences de Luminy-Polytech, CP 925, 13288 Marseille Cedex 09, France; 10 Institute of Microbiology and Genetics, Department of Genetics of Eukaryotic Microorganisms, Georg-August University, Göttingen, Germany; AIT Austrian Institute of Technology GmbH, AUSTRIA

## Abstract

The marine-derived *Scopulariopsis brevicaulis* strain LF580 produces scopularides A and B, which have anticancerous properties. We carried out genome sequencing using three next-generation DNA sequencing methods. *De novo* hybrid assembly yielded 621 scaffolds with a total size of 32.2 Mb and 16298 putative gene models. We identified a large non-ribosomal peptide synthetase gene (*nrps1*) and supporting *pks2* gene in the same biosynthetic gene cluster. This cluster and the genes within the cluster are functionally active as confirmed by RNA-Seq. Characterization of carbohydrate-active enzymes and major facilitator superfamily (MFS)-type transporters lead to postulate *S*. *brevicaulis* originated from a soil fungus, which came into contact with the marine sponge *Tethya aurantium*. This marine sponge seems to provide shelter to this fungus and micro-environment suitable for its survival in the ocean. This study also builds the platform for further investigations of the role of life-style and secondary metabolites from *S*. *brevicaulis*.

## Introduction

Current estimate for numbers of marine fungal species is over 10,000 [1] and this number can even be higher by several folds, if fungi from ocean will be tapped properly ^[^ 2]. Enormous biodiversity of marine fungal isolates is mirrored by the molecular diversity of their secondary metabolites [3–7]. Initial studies of the usages of marine fungi for potential antibiotics was started by Giuseppe Brotzu in 1945, he isolated and cultivated marine-derived *Cephalosporium acremonium* from seawater samples near a sewage outlet in Sardinia [6]. A decade later, Newton and Abraham found that cephalosporin C was the responsible antibiotics [8]. At the end of last decade, over 1000 bioactive compounds have been derived from marine fungi [5–7] with major stockholders are polyketides (40%), alkaloids (20%), peptides (15%), terpenes (15%), prenylated polyketides (7%), shikimates (2%) and lipids (1%) [7]. Despite marine fungi being a potent group of bioactive compound producers, they are not well characterized and still underexplored in terms of biotechnological applications. Hence, there are urgent requirements to focus on the marine fungi and their biosynthetic gene clusters with their capabilities of producing derived bioactive compounds and subsequently pharmaceutical potentials of these compounds. The cyclodepsipeptides scopularides producing fungus *Scopulariopsis brevicaulis* LF580 was isolated from the inner tissue of the marine sponge *Tethya aurantium* collected in the Mediterranean Sea. The two cyclodepsipeptides scopularides A and B produced by *S*. *brevicaulis* LF580 are capable of inhibiting the growth of the pancreatic tumor cell lines (Colo357, Panc89) and the colon tumor cell line (HT29) [9,10]. This data suggest that *S*. *brevicaulis* strain LF580 is capable of producing potentially anti-cancerous compounds, imposing the immediate importance to characterize the genes involved in production of bioactive compounds.

During the last decade, there are rapid advancements in next-generation DNA sequencing (NGS) methods, which opened up a wide facet in discovering and characterization of various aspects of biological science in a cost-effective manner. Major NGS platforms are: Roche GS-FLX 454 pyrosequencer, MiSeq, HiSeq, and Genome Analyzer II platforms (Illumina), SOLiD system (Life Technologies/Applied Biosystems), Ion Torrent, and Ion Proton (Life Technologies), and the PacBio RS II (Pacific Biosciences) [11,12]. NGS methods have become standard approaches for detection of several genes of interests and providing genome wide information [12]. These methods have recently been employed to several fungi including *Sordaria macrospora* [13] and *Pyronema confluens* [14] as well as eukaryotic transcriptomic analyses [15,16].

*S*. *brevicaulis* is an opportunistic fungus, which is capable of growing on several materials and is often found in the soil. Our strain LF580 of *S*. *brevicaulis* was isolated from the inner tissue of the marine sponge *Tethya aurantium* [17]. We set out to perform genomic and transcriptomic approaches regarding the marine-derived *S*. *brevicaulis* strain LF580 to further characterize biosynthetic gene clusters and associated genes with special focus to the gene cluster that produces scopularides. Under normal laboratory conditions, biosynthetic gene clusters are silent and these clusters are activated during stress [18]. Hence, we used UV-based mutant for examining expression patterns of biosynthetic genes of this fungi.

Herein, we present our results from whole genome as well as transcriptome sequencing of the marine sponge-derived *S*. *brevicaulis* strain LF580, which represents the draft genome of this marine-derived species. By comparative genomic analyses, we demonstrate that NRPS1 is responsible for scopularides production. RNA-Seq analyses of the UV-mutant M26 revealed detectable transcripts of about 90% of the genes in the genome, including genes of the NRPS-PKS hybrid cluster (with *nrps1* and *pks2* genes), which is believed to be responsible for scopularides biosynthesis. Finally, we characterized several other features of the *S*. *brevicaulis* genome, such as repeat contents, mating type loci, carbohydrate-active enzymes, MFS-type transporters and performed a protein domain analysis.

## Results & Discussion

### General Genome Features

**Fig 1:**
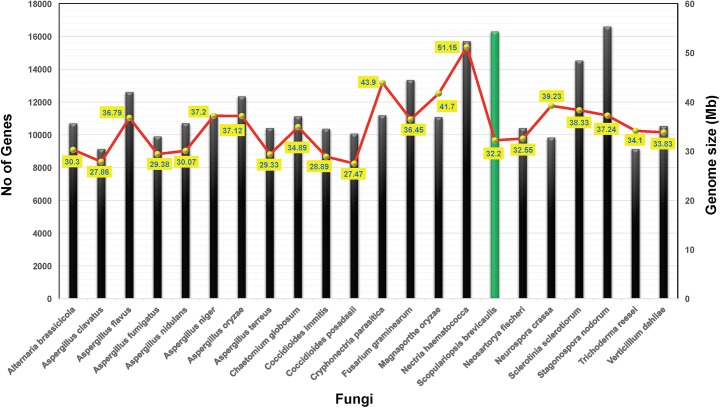
Overview of gene contents and genome size of selected fungi in comparison to *S*. *brevicaulis*.

We have sequenced the genome of the marine-derived *S*. *brevicaulis* using three different genome sequencing methods namely Roche 454, Illumina HiSeq 2000 and ion-torrent. Using Roche 454 pyrosequencing, we achieved a 32.2. Mb genome with N50 equals to 88 kb and 935 contigs, which further joined to form 699 scaffolds with N50 of 116.7 kb. Using short reads of Illumina and Ion-torrent, we achieved smaller N50 (and large numbers of scaffolds) as 67.5 kb (2605) and 26.3 kb (12119), respectively. We performed a hybrid assembly using all these three types of reads, this yielded N50 of 131.8 kb with 623 scaffolds. This corroborates that Roche 454 alone is good performer for fungal genome assembly and combining more than one method is the better choice. This is also reflected by data from a recent genome assembly of the white-rot fungus *Pycnoporus cinnabarinus* [19]. We identified 16,298 putative genes in the assembled genome (**Table 1**). The number of identified genes is rather high when compared to other ascomycetes, which may contain about 10,000 to 12,000 genes (**Fig 1**). The average intron length for this genome is 129.4 bp, which is well within the range of known fungal intron sizes.

**Table 1:** Overview of de novo assembled genome assemblies of S. brevicaulis, produced using Roche 454, Illumina and Ion-torrent sequencing methods.

Assembly characteristics	Roche 454	Illumina HiSeq 2000	Ion Torrent	Hybrid Assembly (Roche 454+Illumina+Ion-torrent)
**Assembled genome size (Mb)**	32.2	32.5	32.6	32.2 Mb
**Number of contigs**	935	3036	35788	661
**Contig N25** * **(** # **Sequences)**	139.9 kb (42)	95 kb (59)	3 kb (1819)	207.5 kb (29)
**Contig N50** * **(** # **Sequences)**	88 kb (114)	59 kb (165)	1.7 kb (5378)	121.2 kb (79)
**Contig N75** * **(** # **Sequences)**	46.4 kb (239)	32.6 kb (346)	0.8 (840)	63.3 kb (166)
**Largest contig (kb)**	342	212.3	14	474
**Average contig (kb)**	34.44	5.1	0.9	49
**Number of scaffold**	699	2605	12119	623
**Scaffold N25** * **(** # **Sequences)**	197.5 kb (30)	109.6 kb (53)	51.4 kb (124)	215 kb (29)
**Scaffold N50** * **(** # **Sequences)**	116.8 kb (83)	67.8 kb (149)	26.3 kb (391)	131.8 kb (76)
**Scaffold N75** * **(** # **Sequences)**	60.3 kb (174)	37.5 kb (310)	8.3 kb (1006)	67.6 kb (158)
**Largest Scaffold (kb)**	474	260	188.3	474
**Average Scaffold (kb)**	46	12.4	2.7	51.7
**%GC content**	54.5	56.3	56.4	56.5
**Number of Genes**	16298			

*Length of the contig/scaffold until which sum of lengths of contigs/scaffolds are reached to 25%, 50% and 75% of total assembled genome size are called N25, N50 and N75 respectively.

#Sequence–Number of sequences (= contigs/scaffolds) in the assembled genome that constitute particular N25 or N50 or N75

### Repeat Elements in *S*. *brevicaulis* Genome

Repeat elements constitute up to 419,240 bp or 1.33% of the assembled genome of *S*. *brevicaulis*. 0.75% of total genome size are tandem repeat sequences, 0.35% are transposable elements (TEs) and 0.20% consisted of low complexity regions (**Table 2 and S1 Table**). Low-complexity regions are regions of biased composition and regions enriched in imperfect direct and inverted repeats [20,21]. Retroelements make up about 0.18% of the *S*. *brevicaulis* genome. Among these retrotransposons with long terminal repeats (LTRs) are in the majorities (0.16%). Class II DNA transposons comprised 0.17% of the genome and the majority of them belong to the Tc1-IS630-Pogo family. Fungal genomes are generally known to possess a low content of only 1–4% of transposable elements [22]. Only a few fungal groups have higher number of repeats, such as several species of dothiodeomycetes [23] and *Tuber melanosporum*, a pezizomycetes species [24]. However, these fungi typically have large expansions of the genome size like *Tuber melanosporum*, which has a genome size of 125 Mb [24]. For further details see a recent review [22].

**Table 2:** Overview of repeat contents of S. brevicaulis genome.

Repeat type	Number of elements*	Percentage of genomic sequence
**Retroelements**	**71**	**0.18**
**Penelope**	**1**	**0.00**
**LINEs**	**11**	**0.01**
**CRE/SLACS**	**3**	**0.00**
**LTR elements**	**60**	**0.16**
**Ty1/Copia**	**10**	**0.00**
**Gypsy/DIRS1**	**50**	**0.16**
**DNA transposons**	**81**	**0.17**
**Tc1-IS630-Pogo**	**68**	**0.16**
**Tourist/Harbinger**	**1**	**0.00**
**Other (Mirage, P-element,Transib)**	**1**	**0.00**
**Total interspersed repeats**		**0.35**
**Small RNA**	**55**	**0.04**
**Satellites**	**1**	**0.00**
**Simple repeats**	**5,151**	**0.75**
**Low complexity**	**1,148**	**0.20**

*Some elements are fragmented hence; numbers are higher in some cases.

### Genome Annotation and Phylogenetic Analysis

**Fig 2:**
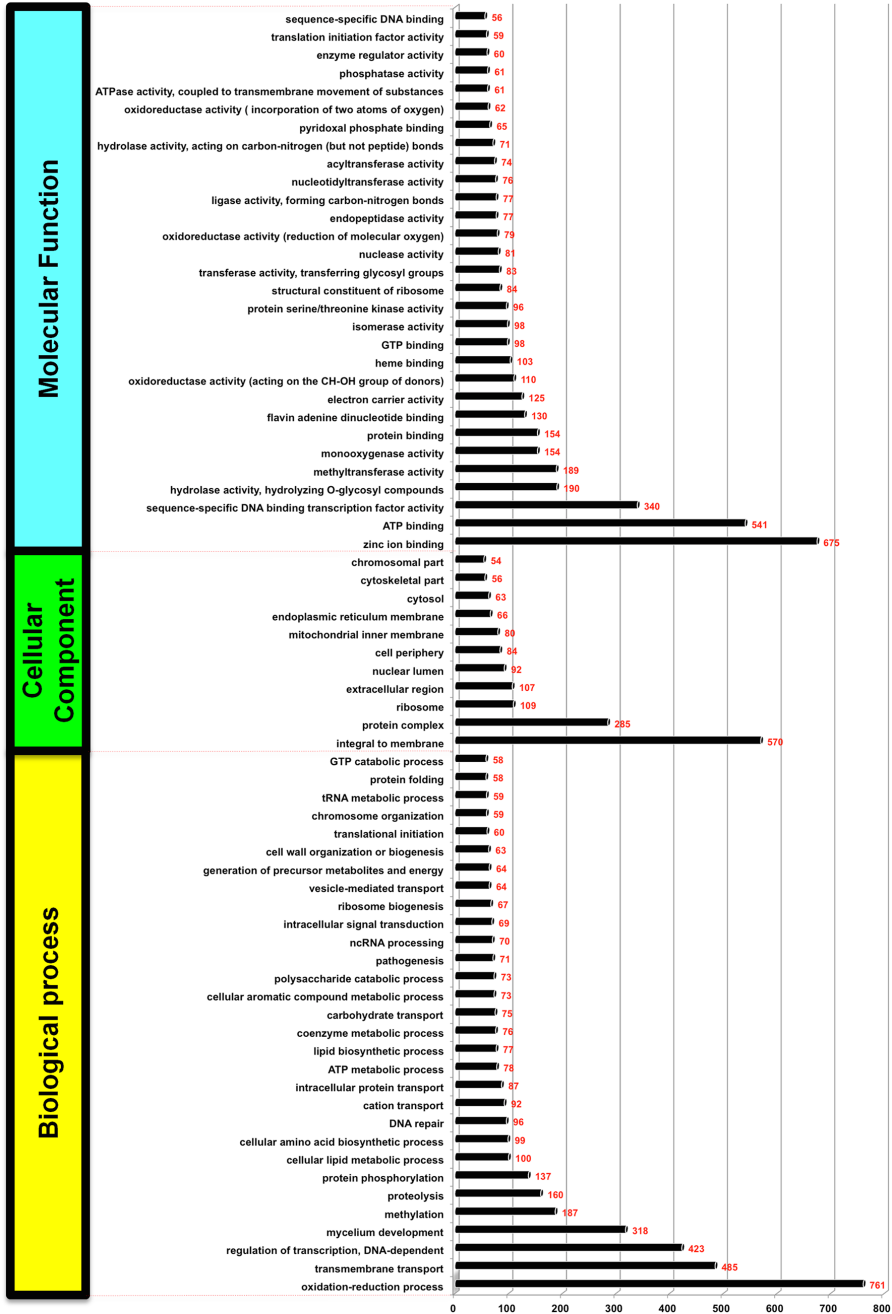
Summary of the three categories of Gene Ontology (GO) terms for *S*. *brevicaulis genes*.

Functional annotation is critical for understanding the genomic data of new species and is supported by Gene Ontology (GO) [25]. GO helps in characterization of genes, transcripts and proteins of many organisms in terms of biological processes (BP), cellular components (CC), and molecular functions (MF) [25]. We have used this method for the functional annotation of *S*. *brevicaulis* proteins using the Blast2GO suite [25]. The derived *S*. *brevicaulis* proteins were assigned to three functional groups based on GO terminology: BP, CC and MF (**S2 Table**). We traced 5,159 proteins to BP terms (**Fig 2**) with the following five top categories: 761 related to oxidation-reduction processes, 485 related to trans-membrane transport, 423 related to regulation of transcription, 318 related to mycelium development, and 187 related to methylation. Under GO annotation of biological processes (BP), we found that *S*. *brevicaulis* is equipped with genes and proteins required for pathogenesis (**Fig 2**). This can be explained by the fact that this opportunistic fungus serves as a pathogen for immune compromised humans and other animals [26].

Similarly, 1,566 proteins were assigned to CC terms (**Fig 2**) with the five top components being: 570 related to integral membrane proteins, 285 related to different protein complexes, 109 related to ribosome proteins, 107 related to extracellular region proteins, and 92 related to proteins of the nuclear lumen. Finally, 4,129 proteins were linked to MF terms (**Fig 2**) with the five top categories as follows: 675 related to zinc ion binding, 541 related to ATP binding, 340 related to sequence-specific DNA binding transcription factors, 190 related to hydrolase activity (hydrolyzing O-glycosyl compounds), and 189 related to methyltransferase activity. All GO terms in these three categories are listed in **S2 Table**.

**Fig 3:**
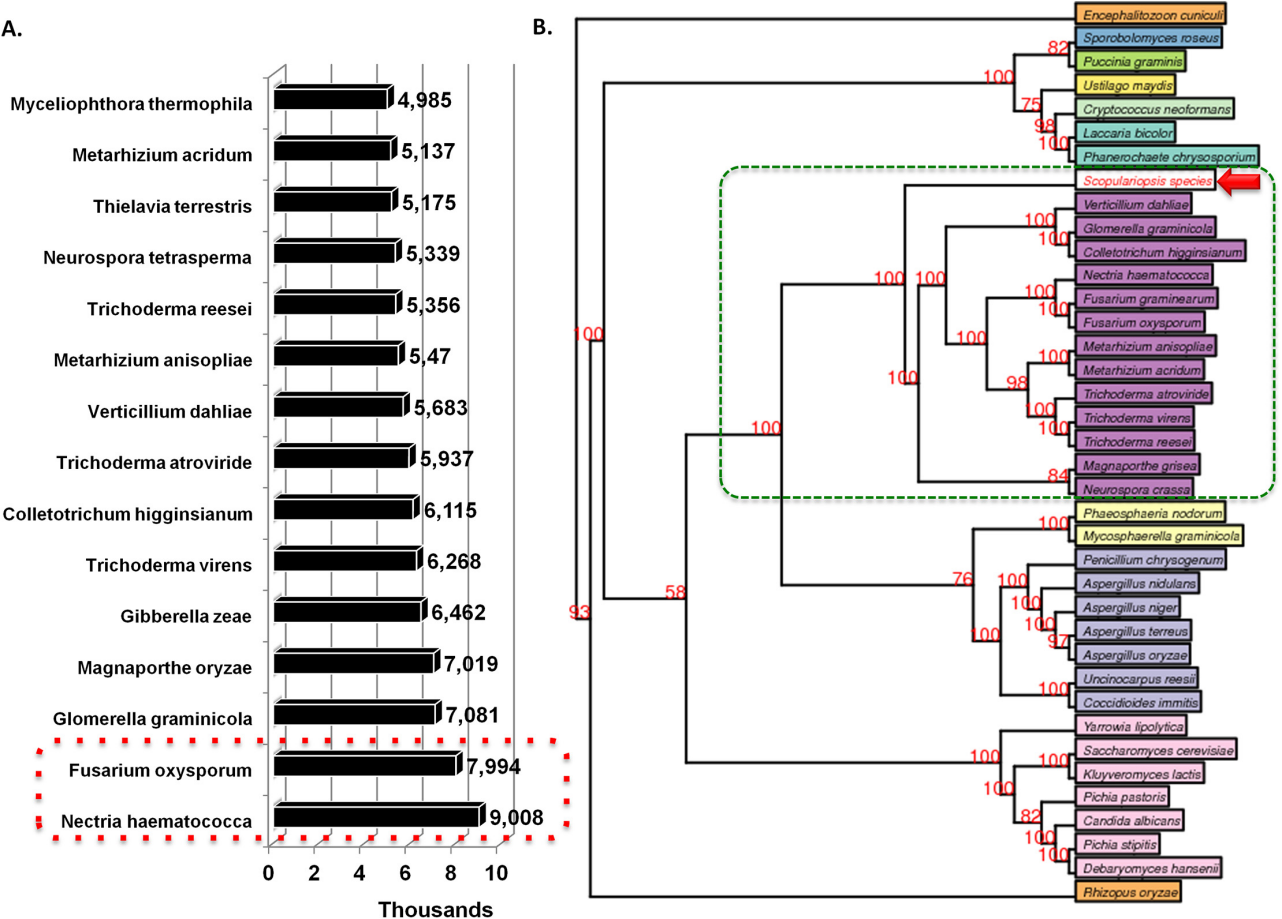
Overview of genome wide localization of *S*. *brevicaulis*. A. Genome-wide homology based annotation data illustrated that *S*. *brevicaulis* is closely related to *Fusarium/Nectria* clade. B. Genome-wide phylogenetic analyses of selected fungi reveal localization of *S*. *brevicaulis*.

During the BLAST2GO based annotation process, we were able to annotate 9,340 genes (57.31%) while 6,958 genes (43.69%) remained non-annotated in this fungus genome as summarized in **S2 Table**. A homology based annotation process suggests *S*. *brevicaulis* belongs to the Sordariomycetes class and it is most closely related to *Nectria* and *Fusarium* species (**Fig 3A**), it does not group within that clade, but seems to be somewhat distinct. To evaluate exact location of this fungus, we performed a genome-wide phylogenetic analysis using the CVtree [27]. We found that *S*. *brevicaulis* has diverged early from other representative Sordariomycetic fungi such as *Verticillium*, *Glomerella*, *Coletotrichum*, *Nectria*, *Fusarium*, *Metarhizium*, *Trichoderma*, *Magnaporthe and Neurospora* (**Fig 3B**).

### Protein Domains of *S*. *brevicaulis*

**Fig 4:**
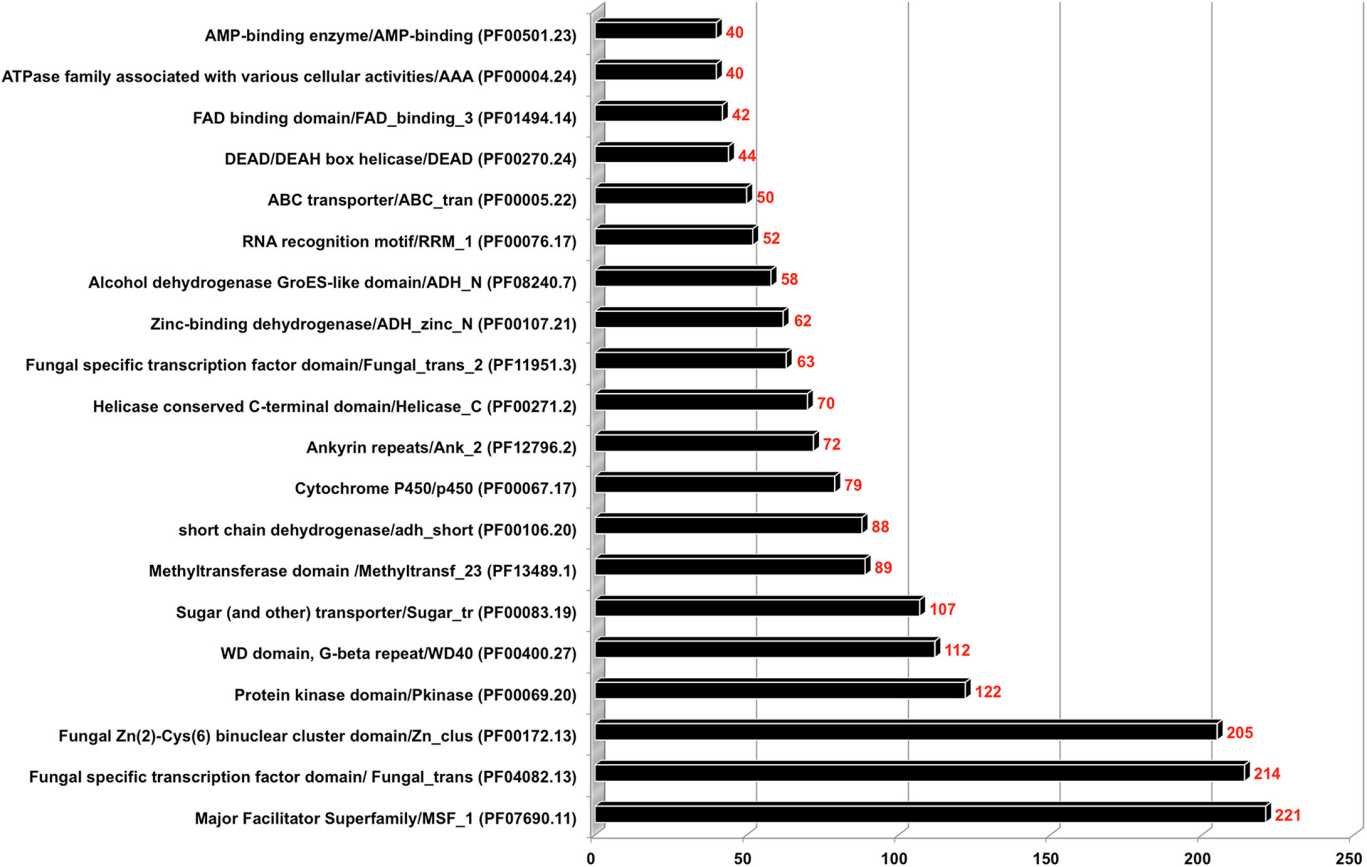
Summary of top 20 Pfam domains of *S*. *brevicaulis* genome encoded proteins. Full details of all Pfam domains are provided in S3 Table.

Protein domains are biochemically independently foldable structural units, which depicting evolutionary conservation with the presence of at least one protein motif. This implies that proteins carrying common domains may have similar functions. Hence, this is an important source for scanning new genomes for putative proteins with similar functions. There are two state of the art databases namely the Pfam [28] and the Interpro [29], both used for protein domain analysis. This analysis is helpful for better annotation of new genomes. We found a total of 10,458 deduced protein sequences of *S*. *brevicaulis* associated with all eukaryotic protein domains (**S3 Table**) and top 20 Pfam domains, which are summarized in **Fig 4**. Additionally, we found two transporter domains in Pfam with 221 proteins harboring a major facilitator superfamily/MSF_1 domain (PF07690.11), and 107 proteins containing a sugar (and other) transporter/sugar_tr domain (PF00083.19). These transporters are generally single-polypeptide secondary carriers involved in transportation of sugars and other small solutes in response to chemiosmotic ion gradients [30,31].

Two transcription factor domains are also in this list with 205 fungal specific transcription factor domain/ Fungal_trans (PF04082.13) and 112 fungal Zn(2)-Cys(6) binuclear cluster domain/Zn_clus (PF00172.13). These proteins serve as transcription regulatory elements. We compared all transcription factor domains, which suggested that these two fungal transcription factor domains are highly expanded in selected ascomycetes (**S4 Table**) as shown previously [32]. We detected 112 G-beta repeat/WD40 (PF00400.27) domains, which may be involved in signal transduction. Additionally, WD40 domains are also regulate fungal cell differentiation processes [33]. We computed comparative protein domain analyses using selected fungal genomes (**S5 Table**). All protein domains of *S*. *brevicaulis* genome are in accordance with known fungal genomes from ascomycetes.

### Plant Biomass Associated Metabolism Evident from Comparative Analyses of Carbohydrate Active Enzymes

The type of association between sponges and fungi and the corresponding ecological function remain unclear and little evidence is available on fungal adaptation to sponges (if any). Studying the carbohydrate-active enzymes (CAZy) profile could provide interesting information on the main families represented in *S*. *brevicaulis* genome and perhaps reveal its substrate preference and its nutritional relationship with the sponge.

Using specialized homology detections and annotation from CAZy database, (www.cazy.org) we identified 478 CAZy genes in *S*. *brevicaulis* genome (**S6 and S7 Tables**). These enzymes are divided into six classes, i.e. 71 auxiliary activities (AAs), 34 carbohydrate esterases (CEs), 50 carbohydrate binding modules (CBMs), 227 glycoside hydrolases (GHs), 81 glycosyl transferases (GTs) and 15 polysaccharide lyases (PLs). *S*. *brevicaulis* contains many candidate enzymes involved in cellulose breakdown as apparent from enzymes from families GH1, GH3, GH5, GH6, GH7, GH12, GH45, with a global diversification similar to other ascomycete fungi able to modify or deconstruct the plant biomass, including the model fungus *N*. *crassa*. *Trichoderma reesei* contains only eleven representatives of families GH5, GH6 and GH7 against 21 for *S*. *brevicaulis*. If we consider hemicellulases and particularly xylanases (families GH10, GH11, GH30 and GH51), galactanases (GH53) or mannanases (GH26), the same picture emerges, *i*.*e*. a similar number of representatives in *S*. *brevicaulis* and *N*. *crassa* but no representative for the entomopathogenic fungus *M*. *anisopliae*. *S*. *brevicaulis* and other plant degrading fungi contain members of the pectinolytic families PL1 (pectin/pectate lyase), PL3 (pectate lyase), PL9 (pectate lyase), PL11 (rhamnogalacturonan lyase), CE8 (pectin methylesterase), CE12 (rhamnogalaturonan acetyl esterase) and GH53 (endo-β-1,4-galactanase). All these data related to glycoside hydrolases (GH) indicate that *S*. *brevicaulis* has developed a metabolism focused on the breakdown of terrestrial plant materials rather than algal or animal biomass.

The carbohydrate portion of land plants is intimately linked to lignin, and auxiliary activities (AA) are needed to give access to GH in order that plant modifying or degrading fungi could penetrate into the cell wall and gain access to the carbohydrate energy source. Considering the AA families acting on lignins, *S*. *brevicaulis* is composed of a poor set of laccase-like oxidases (AA1) and peroxidases (AA2), but with a substantial number of enzymes of the glucose-methanol-choline (GMC) superfamily, i.e. 23 AA3 with one cellobiose dehydrogenase (CDH, AA3_1), 19 putative aryl alcohol oxidases and glucose oxidases (AA3_2), and three alcohol oxidases (AA3_3). In addition, a low number of glyoxal oxidase (AA5), providers of H_2_O_2_ as other members of the GMC family suggest that the fungus does not possess a strong ligninolytic capacity. In contrast, other oxidative enzymes targeting the carbohydrate portion are well represented. For instance, there are four gluco-oligosaccharide oxidases (AA7), and 27 potential members of the lytic polysaccharide mono-oxygenases (LPMO) oxidatively cleaving the glycosidic chains on the crystalline surface of cellulose, chitin or starch (AA9, 11 and 13, respectively). LPMOs create entry points for hydrolytic cellulases, chitinases or amylases. Their recent discovery opened a new route to accelerate biomass degradation in biotechnological applications [34]. Phillips et al. [35] and Bey et al. [36] demonstrated that AA9s and CDH (AA3_1) of *N*. *crassa* and of *Pycnoporus cinnabarinus*, respectively, act in concert to cleave cellulose oxidatively. LPMOs of families AA11 and AA13 recently identified from *N*. *crassa*, *Aspergillus nidulans* and *Aspergillus oryzae* [37–39] are also represented in the *S*. *brevicaulis* genome, suggesting that the fungus could be able to cope with a large variety of plant substrates to degrade.

### Analyses of MFS-Type and Sugar Transporters Also Support Plant Biomass Associated Metabolism

**Fig 5:**
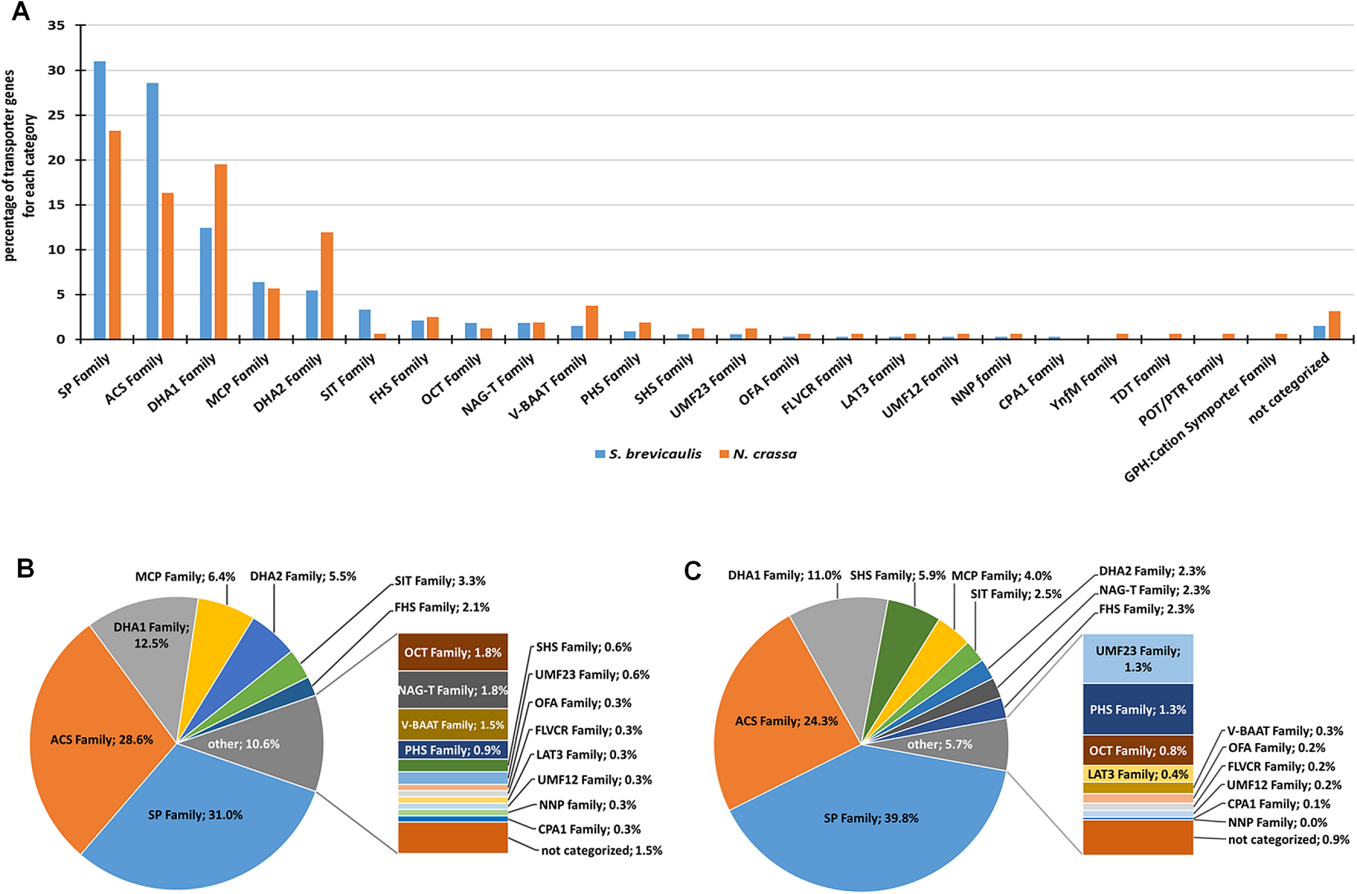
Summary of transporter genes of S. *brevicaulis* and their expression data. **A.** Comparison of MFS type and sugar transporters from S. *brevicaulis* and *N*. *crassa*. This classification is based on TCDB [40] categories. **B.** Distribution of TCDB categories [40] according to the number of classified MFS type and sugar transporters of *S*. *brevicaulis*. **C.** Distribution of TCDB categories [40] according to the combined RPKMs of the assigned MFS type and sugar transporters. The size of each category is presented as percentage of the total number of RPKMs. All categories with less than two percent were grouped together (“other”). The respective values for these categories are presented enlarged in the bar to the right. SP Family (TCDB category: 2.A.1.1): The Sugar Porter Family; OFA Family (2.A.1.11): The Oxalate:Formate Antiporter Family; SHS Family (2.A.1.12): The Sialate:H^+^ Symporter Family; MCP Family (2.A.1.13): The Monocarboxylate Porter Family; ACS Family (2.A.1.14): The Anion:Cation Symporter Family; SIT Family (2.A.1.16): The Siderophore-Iron Transporter Family; OCT Family (2.A.1.19): The Organic Cation Transporter Family; DHA1 Family (2.A.1.2): The Drug:H^+^ Antiporter-1 (12 Spanner) Family; FLVCR Family (2.A.1.28): The Feline Leukemia Virus Subgroup C Receptor Family; DHA2 Family (2.A.1.3): The Drug:H^+^ Antiporter-2 (14 Spanner) Family; YnfM Family (2.A.1.36): The Acriflavin-sensitivity Family; LAT3 Family (2.A.1.44): The L-Amino Acid Transporter-3) Family; V-BAAT Family (2.A.1.48): The Vacuolar Basic Amino Acid Transporter Family; NAG-T Family (2.A.1.58): The N-Acetylglucosamine Transporter Family; UMF12 Family (2.A.1.63): The Unidentified Major Facilitator-12 Family; FHS Family (2.A.1.7): The Fucose: H^+^ Symporter Family; UMF23 Family (2.A.1.75): The Unidentified Major Facilitator-23 Family; NNP Family (2.A.1.8): The Nitrate/Nitrite Porter Family; PHS Family (2.A.1.9): The Phosphate: H^+^ Symporter Family; TDT Family (2.A.16): The Tellurite-resistance/Dicarboxylate Transporter Family; POT/PTR Family (2.A.17): The Proton-dependent Oligopeptide Transporter Family; GPH:Cation Symporter Family (2.A.2): The Glycoside-Pentoside-Hexuronide:Cation Symporter Family; CPA1 Family (2.A.36): The Monovalent Cation:Proton Antiporter-1 Family.

Taking into account the entire CAZyme repertoire, it is clear that *S*. *brevicaulis* has a metabolism capable of break down plant biomass. The same picture emerges when *S*. *brevicaulis* proteins predicted to harbor either a MFS domain (PF07690.11), or a sugar (and other) transporter domain (PF00083.19) are compared to the corresponding transporter complement of *N*. *crassa*, a representative plant biomass saprophyte. *N*. *crassa* has only about half as many transporters encoded in its genome compared to *S*. *brevicaulis* (159 vs. 328 with the same PFam annotations). Yet an overall similar distribution of the transporters can be observed across the categories as defined by the Transport Classification database (TCDB; [40]) can be observed (**Fig 5**). The major categories in both cases are the Sugar Porter family (2.A.1.1), the Anion:Cation Symporter family (2.A.1.14), the Drug:H^+^ Antiporter families 1 and 2 (2.A.1.2 and 2.A.1.3), as well as the Monocarboxylate Porter family (2.A.1.13) (**S8 Table**). Transporters in fungi are notoriously under-characterized, and thus clear annotations are difficult, but the comparison indicates that the two transporter families linked to sugar uptake (2.A.1.1 with 102 vs. 37 members in *S*. *brevicaulis* and *N*. *crassa*, respectively) and the uptake/transport of small charged solutes and metabolites (2.A.1.14 with 94 vs. 26 members) are overrepresented in *S*. *brevicaulis* as compared to *N*. *crassa*, suggesting a broadened substrate spectrum that this fungus is able to utilize. This feature could have been potentially helpful in the transition from *a* soil fungus [26] to a marine sponge habitat. As *Tethya aurantium* (http://www.marlin.ac.uk/index.php, species ID 4450) grows on rocks and stones in the shallow sub-littoral, it is likely that the sponge may have taken up fungal spores drifted from nearby shores. The sponges may have acted as a spore trap or a shelter. Since *S*. *brevicaulis* is able to act as pathogen of humans associated with onychomycosis [26], it may also be able to dwell in a sponge. Therefore, the sponge may have created a suitable micro-environment for a terrestrial fungus that could adapt to the sea salt environment and find nutritional resources. It is known that other fungi from sponges are rather related to fungi from terrestrial sources and are generally able to cope with media containing salt concentration found in the marine environments [41]. Alternatively, it may happen that marine sponge-associated fungi are able to survive without any knowledge of their hosts. It is beyond the scope of this manuscript to explore further details into aspects of fungal-sponge relationships. Nevertheless, our work clears the way for the potential of genomic investigation to study such marine fungal strains.

### Characterization of Gene Content and Expression Using RNA-Seq

The *S*. *brevicaulis* LF580 genome contains over 16,000 genes, which is on the higher side for known ascomycetes (**Fig 1**). It is interesting to see how many of these are expressed in a single condition. To evaluate this status, we extracted RNA of *S*. *brevicaulis* strain M26 growing in WSP30 medium (see Materials and Methods section), which also supports production of Scopularide A and B. We performed RNA sequencing using Illumina HiSeq 2000. Resulting reads were mapped to the putative genes of the assembled *S*. *brevicaulis* genome. A total of 14,724 genes were found to be expressed in this analysis, which represents 90% of the entire gene complement. These expressed genes were classified into 10 tiers based on their reads per kilobase of transcript per million mapped reads (RPKM) values (**Table 3 and S8 Table**). Tier #1 has 120 genes with RPKM values >1000, which accounts for 0.8% of all expressed genes, while 26% (3832 genes) were detected with very low transcript quantities with RPKM values ranging from higher than 0 to lower than 1.0 (marked by red font or blue shade in **S9 Table**, respectively) and these were all placed into tier #10 (non-expressing genes are marked by yellow shade in **S9 Table)**. To further evaluate highly expressing genes in the mutant M26, we examined selected genes and their expression patterns tier-wise according to their RPKM values. In the following, we provide some vignettes of top expressing genes in the UV-mutant M26.

**Table 3:** Top 10 expressed transporter genes.

Tiers	RPKM value	No. of expressed genes	%age of expressed genes	%age of all genes
**Tier #**1	>1000	120	0.8	0.74
**Tier #**2	>250 to <1000	365	2.5	2.24
**Tier #**3	>100 to <250	717	4.8	4.4
**Tier #**4	>50 to <100	1034	7.0	6.34
**Tier #**5	>25 to <50	1615	11	9.9
**Tier #**6	>10 to <25	2579	17.5	15.8
**Tier #**7	>5 to <10	1665	11.3	10.2
**Tier #**8	>2.5 to <5	1365	9.3	8.4
**Tier #**9	1 to <2.5	1442	9.8	8.8
**Tier #**10	>0 to <1	3832	26	23.4

Regarding MFS-type transporters, the accumulated expression per category broadly follows their TCDB classification distribution (compare **Fig 5B and 5C**) with one notable exception. Class 2.A.1.12 (the Sialate:H^+^ Symporter family; dark green), is greatly overrepresented in terms of transcript abundance (only 2 genes, but with 5.9% of total transporter-specific transcript) due to g12790.t1 being the second most highly expressed transporter in the genome (**Table 4**). Homology search by BLAST [42] suggests that g12790.t1 encodes for a carboxylic acid transporter, such as for lactate or pyruvate uptake, which should have been abundant in the rich medium *S*. *brevicaulis* was grown in. An analysis of the remaining genes in the list of top 10 transcribed transporters suggests that these collectively help to satisfy some of the major nutritional requirements of the fungus, such as for carbon and nitrogen as well as vitamins. Sources for these are carbohydrates (hexoses such as glucose, pentoses and other polyols; g14394.t1, g3025.t1, g3159.t1, and g6510.t1), small organic, nitrogenous compounds such as allantoate (g10354.t1), and important nutrients such as the B-vitamins niacin (g116.t1 and g12121.t1) and (potentially) biotin (g10354.t1).

**Table 4:** Overview of expressed genes in the RNA-Seq data of UV-mutant M26 of *S*. *brevicaulis*. Expressed genes are classified into 10 tiers based on their RPKM value.

Gene ID	TCDB Category	RPKM	Putative function based on homology search (NCBI and TCDB BLAST)^1^
g116.t1	2.A.1.14	498.75	Uptake/transport of small, charged organic compounds, such as niacin
g12790.t1	2.A.1.12	373.75	Uptake/transport of carboxylic acids, such as lactate and pyruvate
g9791.t1	2.A.1.1	351.49	Uptake/transport of hexoses or their polyol derivatives, such as myo-inositol
g14394.t1	2.A.1.1	283.53	Putative glucose transporter
g3025.t1	2.A.1.1	229.95	Putative pentose transporter
g3159.t1	2.A.1.1	210.41	Putative glucose transporter
g7247.t1	2.A.1.2	209.82	unknown
g10354.t1	2.A.1.14	207.97	Uptake/transport of small, charged nitrogen-containing compounds, such as allantoate
g12121.t1	2.A.1.14	202.96	Uptake/transport of small, charged organic compounds, such as niacin
g6510.t1	2.A.1.1	195.59	Putative glucose transporter

^1^ for details, see **S8 Table**

Hydrophobins are morphogenetic, small mass (≤20 kDa) secreted hydrophobic fungal proteins [43]. *S*. *brevicaulis* has three hydrophobin genes, namely SbreHPB1 (g5510.t1), SbreHPB2 (g7216.t1), and SbreHPB3 (g15602.t1). Two hybrophobins (SbreHPB1 and SbreHPB2) were expressed in the top expression tiers #3 and #1 with RPKM values of 101.6 and 1035, respectively. In contrast, SbreHPB3 had lower expression values (60 RPKM) and was classified into the tier #4. Other recent studies also indicated that fungal hydrophobins have different expression patterns under abiotic and biotic stresses, in which adherence mechanisms are influenced [44,45]. Furthermore, common numbers of hydrophobins are generally 2–10 per fungus with no apparent increase in copy number in fungi of marine origin [46].

In summary, transcripts for about 90% of the genes in this genome could be detected. This rather high value indicates that *S*. *brevicaulis* has a higher number of expressed genes than most other Ascomycetes.

### Overview of Bioactive Compounds Encoding Genes

**Fig 6:**
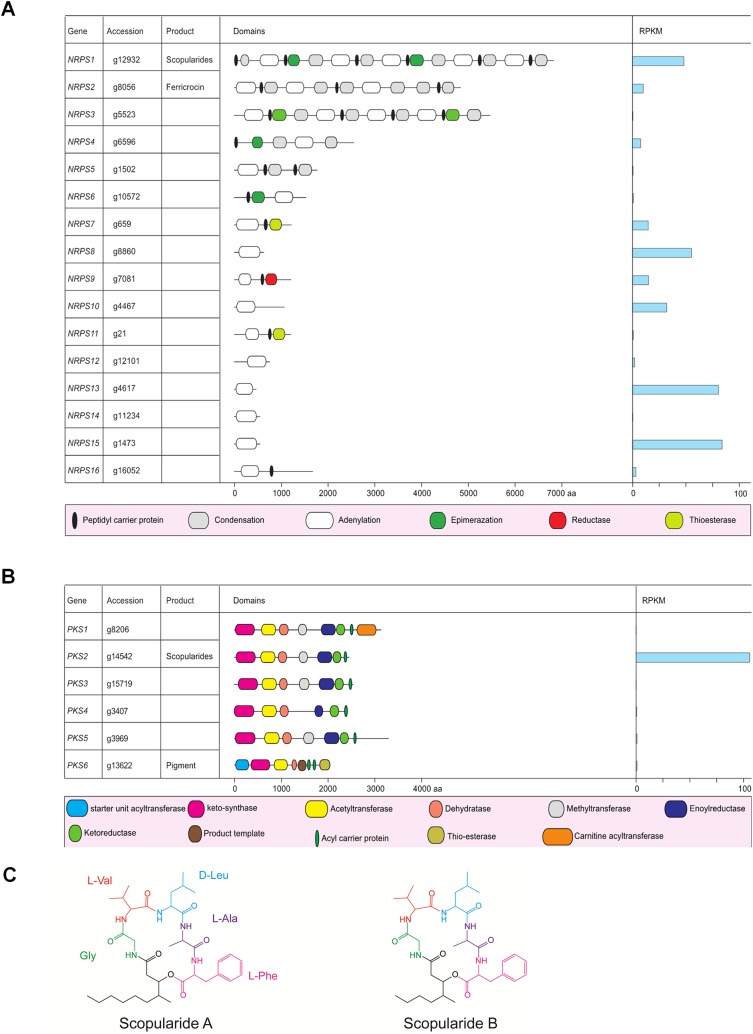
Overview of protein domains and expression values of putative NRPS and PKS encoded in the *S*. *brevicaulis* genome. A. Summary of NRPS genes reveals three multimodular NRPS and thirteen non-modular NRPS genes. Both of these NRPS genes are expressed in RNA-seq data. B. List of full-length polyketide synthetases (PKS) genes and corresponding protein domain organization. C. Proteins encoded by the nrps1 and psk2 gene are capable of producing scopularide, which has two forms A and B.

**Fig 7:**
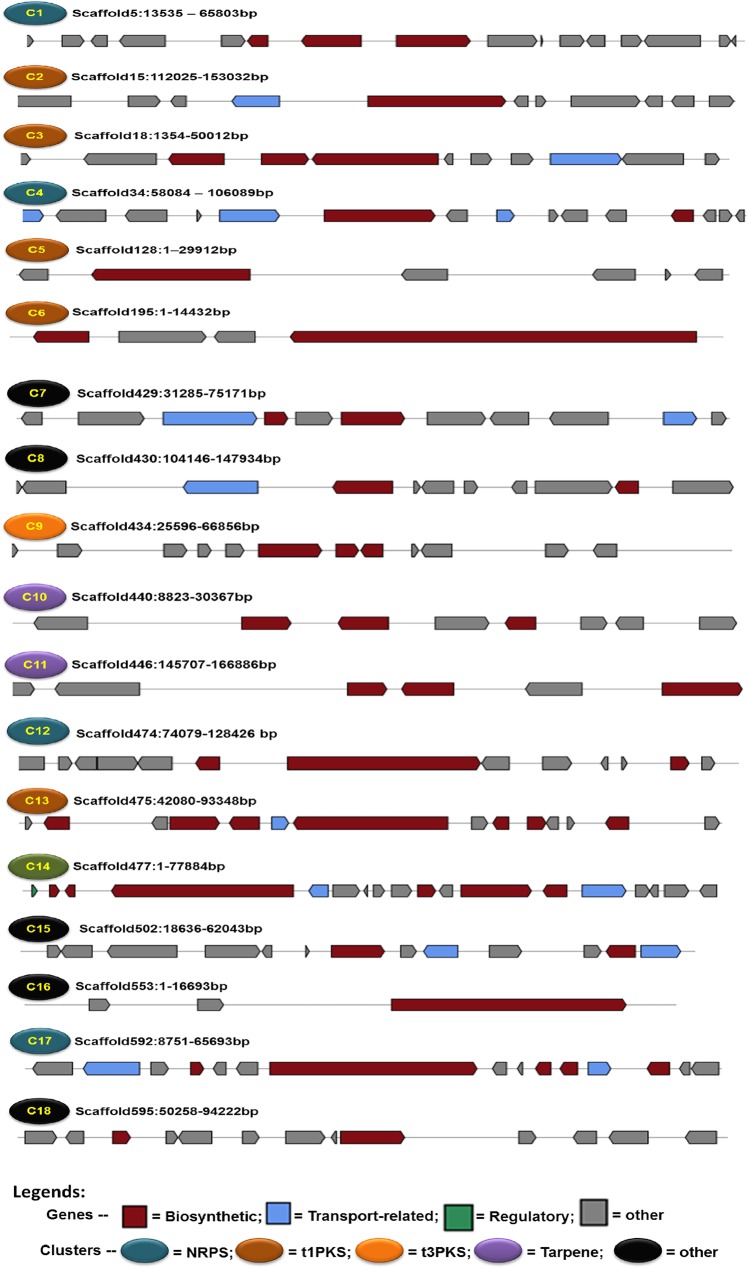
Summary of 18 identified gene clusters responsible for the production of different bioactive compounds in the assembled genome of *S*. *brevicaulis*. Gene clusters are numbered as C1-C18 followed by scaffold and positions on the scaffold and different colors are illustrating different types of clusters. Biosynthetic genes are key gene (like either nrps or pks and so on) and main supporting genes such as a cytochrome P450 gene as per antiSMASH [48] guidelines. Similarly, other genes are any other gene in the cluster, which are not key genes, regulatory (such as transcription factor or suppressor) and transporters (such as ABC transporter) and are marked in grey shade.

**Fig 8:**
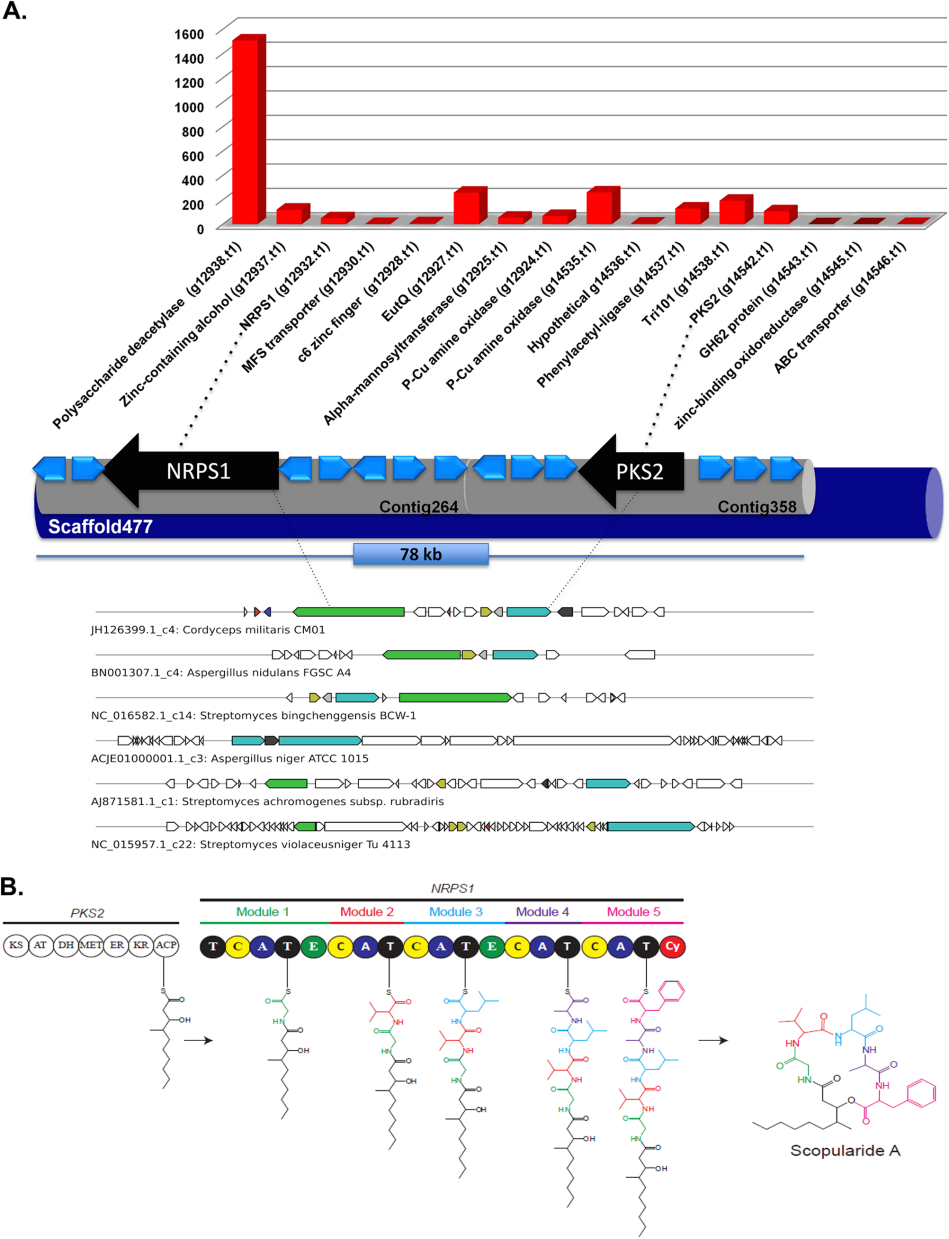
Overview of scopularide producing mechanism. **A. Summary of scopularide producing cluster of NRPS1/PKS2 of *S*. *brevicaulis*.** This cluster is localized on the scaffold477 in a region of 78 kb at the 3’ end of this scaffold (scaffold size 280kb). This cluster is active as different flanking genes (marked in blue) are expressed during UV-mutagenesis based RNA-Seq experiment. Top homologs clusters are found in both fungi and bacteria, which hints that this clusters might have originated via horizontal gene transfers from bacteria. P-Cu amine oxidase—Peroxisomal copper amine oxidase; Tri101—Trichothecene 3-o-acetyltransferase; EutQ—Ethanolamine utilization protein like EutQ. **B. Schema of generation of scopularide *using NRPS1 and PKS2 of S*. *brevicaulis*.** Modified from *Lukassen et al*. [51].

The *S*. *brevicaulis* genome has 16 genes encoding for non-ribosomal peptide synthetases (NRPSs) (**Fig 6**) with three NRPS genes (NRPS1-3) encoding enzymes with a multi-modular organization with more than one condensation domain. This modular architecture is known to be specific for fungal NRPSs [47]. The domain organization of putative NRPS and PKS proteins is shown in **Fig 6**. Additionally, we identified six full-length polyketide synthase genes (PKSs), one fatty acid synthase (FAS) gene and three putative terpene encoding genes in the genome (**Table 5**). Additional single domain enzymes such as reductases and cytochrome P450 monooxygenases were also identified but not taken into further consideration. All these genes are localized into 18 different clusters (**Fig 7 and S2 Fig**), which include four NRPS clusters, six PKS clusters, and five other clusters that have NRPS6-NRPS16 genes. Since the encoded NRPSs of these genes are not modular in nature, these are placed separately by the AntiSMASH tool [48] in comparison to other clusters. A single cluster was identified on the scaffold477, which possesses NRPS1 and PKS2 genes in the N-terminal 78 kb region (**Fig 8**), which is composed of the contig264 and contig358. Corresponding clusters of supporting genes and their expression values are shown in **Fig 8**. Our data indicate that this gene cluster, which is involved in the scopularide production and indeed, it is actively expressed under conditions supporting scropularides A and B production [49,50]. The *nrps1* gene (g12932) is the best candidate gene to be responsible for production of the cyclic lipopeptide scopularide [51], which consists of five amino acids (glycine, L-valine, D-leucine, L-alanine and L-phenylalanine), and a reduced carbon chain [52]. Its production scheme is shown in **Fig 8B**. The reduced carbon chain (3-hydroxy-methyldecanoyl) may be derived from the product of the *pks2 gene*. This is further supported by the fact that the two genes (nrps1 and pks2) are localized on a single cluster on the scaffold477. This cluster has a high degree of similarity with clusters in the genomes of *Cordyceps militaris* (JH126399.1), *Aspergillus nidulans* FGSC A4 (BN001307.1), *Streptomyces bingchenggensis* (NC_016582.1), *A*. *niger* ATCC 1015 (ACJE01000001.1), and *Streptomyces achromogenes* subsp. rubradiris (AJ871581.1) (**S2 Fig**). **S2 Fig** depicts details of these clusters with information about homologous clusters in either fungi or bacteria. Obviously, most of these 18 clusters have homologous in closely related ascomycetes, as shown in **Fig 3** on a phylogenetic scale. However, we could not identify in other fungal genomes any homologous for clusters 5 and 16 (**S2 Fig**). However, some clusters exhibit similarities to bacterial counterparts, which is especially true for the cluster 5 (**S2E Fig**) with a homolog in only *Streptomyces violaceusniger* (NC_015957.1). This may suggest horizontal gene transfer from bacteria to fungi. Indeed, horizontal gene transfer is considered to be a major source of metabolite diversity in fungi [53]. The *nrps2* gene (g8056) encodes an enzyme which is a homologue of the synthetase responsible for production of the iron-chelating siderophore ferricrocin (*SidC*) [54], found in numerous fungi [55]. The third multi-modular *NRPS*, encoded by *nrps3* (g5523) contains four adenylation domains, but the product is currently unknown. The gene was not expressed under the examined conditions and BLASTP analyses did not identify orthologs with known products.

**Table 5:** Summary of secondary metabolite encoding genes in the *S*. *brevicaulis* genome.

Putative genes encoding key protein for bioactive compound	*Number*
Non-ribosomal peptide synthetase (NRPS)	16
Polyketide synthase (PKS)	6*
Fatty acid synthase (FAS)	1
Tarpenes	3

*Only full length PKS genes are considered.

Five of the six PKS proteins (*PKS1*-*5*) contained the reducing domains dehydratase, enoylreductase and ketoreductase. The only actively expressed PKS gene was *pks2* (g14542), which has possible orthologs in *Aspergillus nidulans* (AN2547), *F*. *graminearum* (*PKS6*; FGSG_08208) and *F*. *pseudograminearum* (*PKS40*, FPSE_09183). The encoded PKSs are involved in production of the lipopeptides emericellamide, fusaristatin and W493, respectively [56,57], each consisting of a reduced carbon chain provided by the PKSs, which is requited by NRPSs together with three to seven amino acids. The resulting product is then released by the NRPS by cyclization. The *pks6* gene (g13622) on the other hand has a non-reducing PKS protein product and BLASTP analysis against GenBank showed that it is shares similarities with the mycelium pigment synthase and shares 71% identity to a PKS (VDAG_00190) that has been proposed to be involved in the biosynthesis of melanin in *Verticillium dahlia* [58]. Hence, *PKS6* could be involved in pigment biosynthesis in *S*. *brevicaulis*. These pks genes are type I PKS genes and are localized in 5 different clusters (**Fig 7 and S2 Fig**). Cluster 9 is the only cluster (**Fig 7 and S2 Fig**), which can lead to type III PKS, which might be responsible for the production of chalcone and stilbene synthase as the key enzyme shows 70% identities with homologous gene in the *Colletotrichum higginsianum* (GenBank, ID CCF34076.1). We also identified three genes encoding aristolochene synthase (g9860.t1), geranylgeranyl diphosphate synthase (g13546.t1) squalene synthase (g5738.t1) forming two clusters (**Fig 7 and S2 Fig**) on the scaffolds scaffold440 and scaffold446.

At current the secondary metabolite products produced by many of these proteins are unknown as it is typical for many fungi studied. As genome sequencing of fungi has become affordable we expect more and more fungal genomes being available in the public databases, which will lead into a better picture of homologous gene clusters and their final products. This opens opportunities for other researchers for utilizing genome wide information of this fungus to explore the potentials of these genes and their clusters. In addition this analysis, a separate study was carried out for characterization of scopularide producing proteins using iTRAQ-based proteomics analysis [59].

### *S*. *brevicaulis LF580* Is a Mating Type MAT1-1 Strain

**Fig 9:**
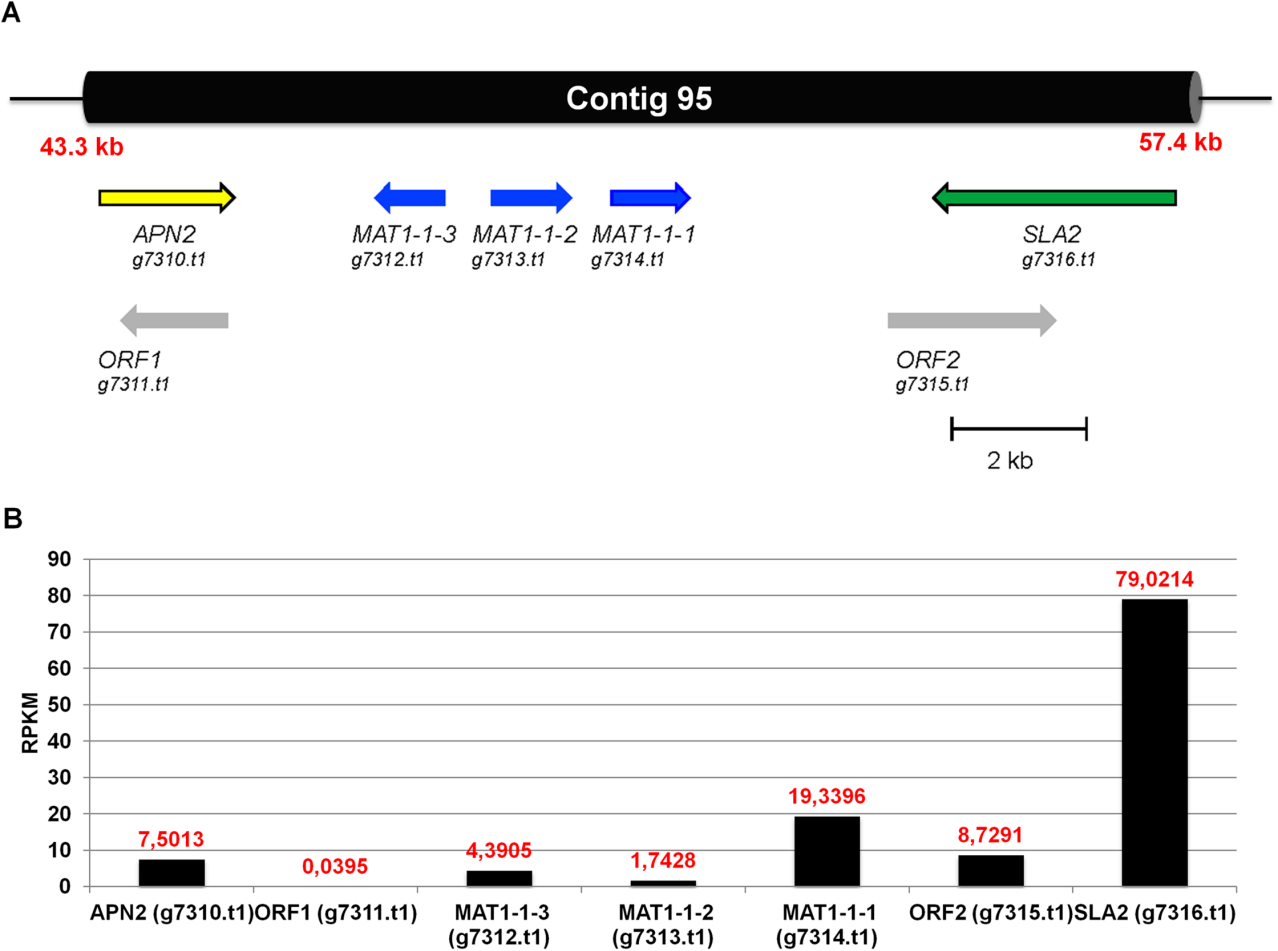
Overview of the mating-type locus MAT1-1 of *S*. *brevicaulis*. A. The mating-type locus MAT1-1 of *S*. *brevicaulis* is localized on contig 95. The positioning and transcriptional direction of the mating-type genes (blue) is indicated by an arrow. Flanking genes *APN2* and *SLA2* are shown in yellow and green, respectively. Two predicted ORFs are indicated in grey. B. Expression profiling of genes conserved in the mating-type locus MAT1-1 of *S*. *brevicaulis*.

Mating type (MAT) locus governs sexual reproduction in fungal kingdom by possessing key transcriptional regulators that facilitates the cell identity and fate [60]. We detected three MAT1-1 specific ORFs, *MAT1-1-1* (g7314.t1), *MAT1-1-2* (g7313.t1) and*MAT1-1-3* (g7312.t1) on contig 95 in the *S*. *brevicaulis* genome (**Fig 9**) with help of several parameters for homology detection using BLAST suite [42]. The MAT1-1 specific genes are flanked by the *SLA2* and *APN2* genes on contig 95 of *S*. *brevicaulis* (**Fig 9A**), and these two genes are frequently found close to the MAT loci in filamentous ascomycetes [61–64]. In addition, two putative ORFs (gi7311.t1 and gi7315.t1) are predicted in a reverse orientation of *APN2* and *SLA2*, respectively. However, a BLASTP search with these two ORFs gave no significant hit in the databases.

Upon scanning expression profiling based on RNA-seq data, we found that all of the mating type genes of *S*. *brevicaulis* are expressed. The *MAT1-1-1* gene has highest expression among three mating genes, which is followed by *MAT1-1-3* and *MAT1-1-2* (**Fig 9B**). The flanking gene *SLA2* gene has a particular high expression, which is 10-fold higher in comparison to the *APN2* gene. Overall, we report that conserved genes of the mating type loci of *S*. *brevicaulis* are expressed.

Overall, the presence of three MAT1-1 genes and absence of MAT1-2 gene in the *S*. *brevicaulis genome* corroborate that *S*. *brevicaulis* LF580 is a MAT1-1 strain. Additionally, all three MAT1-1 genes appear to be functional genes, because these genes shown expression profiles in the RNA-Seq datasets.

## Conclusions

Our article presents the draft assembly of the *S*. *brevicaulis* strain LF580 genome isolated from marine environment. Using three different sequencing methods, the genome was assembled with genome size of 32.2 Mb harboring 16,298 putative genes. We identified 18 gene clusters responsible for secondary metabolite production, which appear to express secondary metabolite enzymes. This includes a cluster with NRPS1 and PKS2 genes, which together synthesize scopularides with anticancerous properties. In summary, by combining genomic and transcriptomic data, we have compiled new genetic and expression information for a marine-derived strain of *S*. *brevicaulis*. Moreover, we analysed the obtained genome data for clues explaining the necessary life style changes.

## Methods

### Collection of Fungal Strain, Cultivation, and DNA Isolation

*S*. *brevicaulis* LF580 strain was cultivated as previously described [49]. The strain was obtained from the fungal collection of the Kiel Center for Marine Natural Products as cryo-conserved material. Originally, this strain was isolated from the inner tissue of the marine sponge *Tethya aurantium*. This fungus was cultivated on solid WSP30 medium, which is a variant of Wickerham-medium (with composition as following 1% glucose, 0.5% soy peptone, 0.3% malt extract, 0.3% yeast extract, 3% NaCl) [65]. *S*. *brevicaulis* M26 [47] was provided by Linda Paun (Kiel). Genomic DNA from *S*. *brevicaulis* was prepared by following a modification of previously published methods [66,67]. Mycelium was frozen in liquid nitrogen, pulverized, and incubated in equal volumes of lysis buffer (10 mM Tris-HCl, 1 mM EDTA, 100 mM NaCl, 2% SDS, pH 8.0), After centrifugation, the supernatant was treated with RNase, and afterwards with an equal volume phenol/chloroform (1:1).

### Genome Sequencing Using Three Different Methods

Roche 454 sequencing was performed with 20 μg genomic DNA at Macrogen (Korea). This provided 631 Mb 454 reads with an average read length of 432.5 bp (**S3 Fig**). Illumina sequencing was performed using Illumina HiSeq™ 2000 with 20 μg genomic DNA at the Macrogen (Korea). This yielded in 2.5*10^8^ Illumina reads with an average length of 101 bp (**S10 Table**). Ion-torrent sequencing was carried out with 20 μg genomic DNA at Genotypic Technology (Bangalore, India) and 630 Mb of Ion-torrent reads were generated with average length of 119 bp (**S11 Table**). The whole genome sequencing and RNA-Seq data for *S*. *brevicaulis* is publically available using BioSample accession ID: SAMN03764504 and corresponding BioProject accession ID: PRJNA288424.

### Genome Assembly, Repeat Detection, Gene Prediction and Annotation Analyses

Roche 454 reads were assembled into contigs using Newbler assembler [68]. Several Genome assemblies were performed using de Brujin graph based method by de novo assembler in the CLCBio Genomic workbench [69] using generated reads of Illumina, and Ion-Torrent and all reads for hybrid assembly. Scaffolding of contigs generated by respective assemblies was carried out using genome finishing module of CLCBio Genomic workbench [69]. The Repeat elements were predicted using RepeatMasker and RepeatProteinMasker software programs (Smit, AFA, Hubley, R & Green, P. RepeatMasker Open-4.0.0 1996–2013 http://www.repeatmasker.org) using the fungal transposon species library (database version 20120418) as input. Gene prediction was performed using Augustus gene prediction tool using *Asperigillus niger* as training dataset. This prediction was compared with other genome prediction tools. Predict genes were annotated using BLAST homology searches [42] with an E-value cutoff of 1e^−3^, supported by BLAST2GO tool [25]. Predicted coding regions were annotated using BLAST [42] with comparing the Kyoto Encyclopedia of Genes and Genomes (KEGG) [70], Swiss-Prot, TrEMBL, Gene Ontology (GO), and non-redundant (NR) databases.

### Genome-Wide Phylogenetic Relationship

In order to confirm the phylogenetic position of species under study we reconstructed a phylogenetic tree using the CVtree [27]. CVtree is an alignment free composition vector tree based method and hence does not require selection of specific genes for phylogeny reconstruction. The only parameter required by the method is k, that was set to 7 [71]. We used the fully predicted proteomes of 67 fungi, and the choanoflagellate *Monosiga brevicollis* as an outgroup [72]. Bootstrap scores for phylogeny were calculated as in [73] by randomly sampling the proteome of each species, with replacement, to create a novel perturbed proteome for each of the 100 bootstrap runs. A representative subset of the 68 species was plotted using APE package [74] within the R computing environment [75].

### Protein Domain Estimation

Predicted proteins of this genome were scanned to all known Pfam (version 27) [28] and Interpro (version 43) [29] protein domains collections, respectively. Pfam domains were predicted using HMMER 3.0 [76], removing overlapping clans. In order to compare protein coding gene content across fungal species we constructed a python script to carry out the following tasks. Interpro database [29] was searched for Pfam [28] identifiers corresponding to Interpro identifiers of interest. For each Pfam [28] identifier pfam_scan.pl (ftp://ftp.sanger.ac.uk/pub/databases/Pfam/Tools/), a wrapper for HMMER 3.0 [76], was run to find the matching proteins in the genomes of interest. To analyse subfamily structure of each Pfam family’s member proteins in the genomes, the corresponding protein sequences were collected and mcl clustered [77]based on the E-value matrix of all-vs-all BLASTP [42]. The e-value matrix was tresholded prior to clustering [78]. The mcl clustering has a single major parameter that defines the granularity of the clustering i.e. the inflation value. mcl clustering was run over the range of possible inflation values. For each inflation value, a sensitivity and specificity was calculated for the clustering as previously described [32,79]. In order to calculate these, other secondary Pfam matches were determined for the member proteins of the Pfam under study and the most variable secondary Pfam selected for sensitivity and specificity calculations. Sensitivity and specificity were centred and the inflation value corresponding to their minimum difference selected to get a single subfamily clustering for each Pfam. A R-script using the APE package [74] within the R computing environment [75] was used to plot and process the result tables. The program code for the analysis is available at https://github.com/fahad-syed/ProSol.git.

### Identifications and Classifications of CAZyme Domains

All putative proteins were compared to the entries in the CAZy database [80,81] using BLASTP [42]. The proteins with E-values smaller than 0.1 were further screened by a combination of BLAST searches against individual protein modules belonging to the following classes auxiliary activities (AA), glycoside hydrolases (GH), glycosyltransferases (GT), polysaccharide lyases (PL), carbohydrate esterases (CE) and carbohydrate-binding modules (CBM) in the CAZy database (http://www.cazy.org/). HMMER 3.0 [76] was used to query a collection of custom-made hidden Markov model (HMM) profiles constructed for each CAZy family. All identified proteins were then manually curated and whenever possible, assigned to a subfamily within a family.

### Classification of MFS-Type and Sugar Transporters

For a more precise classification of the *S*. *brevicaulis* genes annotated according to Pfam as MFS-type or sugar transporters (328 genes total), the Transporter Classification Database (TCDB) was used [40]. In addition, the 159 transporter genes of *N*. *crassa* with the same Pfam annotations were also classified using TCDB [40]. To this end, sequence similarity searches were performed against the TCDB for each gene using BLASTP [42] with default parameters. To ensure a certain level of stringency, only E-value of 1e^-10^ and below were considered as reliable hits. If a BLAST [42] search met these preconditions, the corresponding gene was classified into the same category as the TCDB homolog with the best e-value. When the E-value threshold was exceeded for all TCDB results, the gene was not categorized. Some TCDB results exhibited low e-values and diverse categories. In these cases, the respective genes were flagged as uncertain, but still categorized for further analysis.

Moreover, the ten most highly expressed genes were further analyzed by performing a BLASTP [42] sequence similarity search against the RefSeq database (NCBI) with default parameters to identify homologs not present in TCDB with a descriptive annotation.

### RNA Isolation, Sequencing and RNA-Seq Analyses

Cultivation of fungal strain M26 was done in WSP-30 medium for 7 days at 200 rpm in the dark. RNA was isolated using previously known methods for RNA isolation [13,14,66]. RNA sequencing was performed using Illumina HiSeq™ 2000 at the Beijing Genome Institute (BGI) (Shenzhen, China). A total of 17,452,507 illumina reads were obtained for the *S*. *brevicaulis*. Raw reads were mapped to predicted genes using RNA-Seq mapping tool of CLC Bio Genomic workbench [69] and relative expression levels were calculated as Reads Per Kilobase of transcript per Million mapped reads (RPKM).

### Detection of Bioactive Encoding Genes and Their Clusters

Initially, putative genes that encoding for proteins which produce bioactive compounds are identified using BLAST [42] with an E-value < 1e^−3^. Subsequently, this genome was analysed using SMURF [82] and antiSMASH [48] for putative clusters and further examined by manually coupled with RNA-Seq data. The functional domains of PKSs and NRPSs were identified as previously described [83], using a combinations of tools namely antiSMASH [48], NCBI Conserved Domain Database [84], InterPro [29] and the PKS/NRPS Analysis Web-site [85].

## Supporting Information

S1 Fig Summary of ribosomal proteins expressed in top 3 tiers in the mutant M26. (JPG)

S2 Fig Summary of 18 secondary metabolite clusters from *S*. *brevicaulis* and their homologs in different fungal and bacterial genomes. Two clusters namely cluster 11 and 16 have no homologs in known fungal genomes.
(PDF)

S3 Fig Summary of Roche 454 reads. (PDF)

S1 Table Overview of repeat contents in the *S*. *brevicaulis* genome. (XLSX)

S2 Table Summary of genome annotation of *S*. *brevicaulis* genome (XLSX)

S3 Table Pfam domain annotation of *S*. *brevicaulis* genome wide peptides. (XLS)

S4 Table Overview of transcription factors derived from protein domain analysis. (XLSX)

S5 Table Comparison of protein domains of *S*. *brevicaulis* with selected fungi. (XLS)

S6 Table Overview of CATyome of *S*. *brevicaulis*. (XLSX)

S7 Table Comparisons of CATymes of *S*. *brevicaulis* and selected fungi. (XLSX)

S8 Table Overview of transporter genes of *S*. *brevicaulis*. (XLSX)

S9 Table Summary of transcriptomics of mutant M26 of *S*. *brevicaulis* generated by UV mutagenesis generated using Illumina based RNA sequencing. Tiers are defined in the Table 4.
(XLS)

S10 Table Quality control and base reports of Illumina reads. Fungi1—Scopulariosis.
(XLSX)

S11 Table Quality control and base reports of Ion-Torrent reads. (XLSX)

## References

[1] Jones EBG (2011) Are there more marine fungi to be described? Botanica Marina 54: 343–354.

[2] Mora C, Tittensor DP, Adl S, Simpson AGB, Worm B (2011) How many species are there on Earth and in the ocean? PLoS biology 9: e1001127–e1001127. 10.1371/journal.pbio.1001127 21886479 PMC3160336

[3] Konig GM, Kehraus S, Seibert SF, Abdel-Lateff A, Muller D (2006) Natural products from marine organisms and their associated microbes. Chembiochem 7: 229–238. 16247831 10.1002/cbic.200500087

[4] Ebada SS, Proksch P (2013) Bioactive secondary metabolites from marine-derived fungi In: Kim SK, editor. Marine Pharmacognosy:Trends and Applications: CRC Press Taylor & Francis Group, LLC, Boca Raton pp. 27–51.

[5] Saleem M, Ali MS, Hussain S, Jabbar A, Ashraf M, et al (2007) Marine natural products of fungal origin. Nat Prod Rep 24: 1142–1152. 17898901 10.1039/b607254m

[6] Bugni TS, Ireland CM (2004) Marine-derived fungi: a chemically and biologically diverse group of microorganisms. Nat Prod Rep 21: 143–163. 15039840 10.1039/b301926h

[7] Rateb ME, Ebel R (2011) Secondary metabolites of fungi from marine habitats. Nat Prod Rep 28: 290–344. 10.1039/c0np00061b 21229157

[8] Newton GG, Abraham EP (1955) Cephalosporin C, a new antibiotic containing sulphur and D-alpha-aminoadipic acid. Nature 175: 548 14370161 10.1038/175548a0

[9] Yu Z, Lang G, Kajahn I, Schmaljohann R, Imhoff JF (2008) Scopularides A and B, cyclodepsipeptides from a marine sponge-derived fungus, Scopulariopsis brevicaulis. J Nat Prod 71: 1052–1054. 10.1021/np070580e 18412398

[10] Imhoff JF, Kajahn I, Lang G, Wiese J, Peters A (2010) Production and use of antimumoral, antibiotic and insecticidal cyclodepsipeptides (WO 2010/142258).

[11] Metzker ML (2010) Sequencing technologies—the next generation. Nat Rev Genet 11: 31–46. 10.1038/nrg2626 19997069

[12] Culligan EP, Sleator RD, Marchesi JR, Hill C (2013) Metagenomics and novel gene discovery: Promise and potential for novel therapeutics. Virulence 5: 1–14. 24317337 10.4161/viru.27208 PMC3979868

[13] Nowrousian M, Stajich JE, Chu M, Engh I, Espagne E, et al (2010) De novo assembly of a 40 Mb eukaryotic genome from short sequence reads: Sordaria macrospora, a model organism for fungal morphogenesis. PLoS genetics 6: e1000891–e1000891. 10.1371/journal.pgen.1000891 20386741 PMC2851567

[14] Traeger S, Altegoer F, Freitag M, Gabaldon T, Kempken F, et al (2013) The Genome and Development-Dependent Transcriptomes of Pyronema confluens: A Window into Fungal Evolution. PLoS Genetics 9: e1003820–e1003820. 10.1371/journal.pgen.1003820 24068976 PMC3778014

[15] Kumar A, Congiu L, Lindström L, Piiroinen S, Vidotto M, et al (2014) Sequencing, De Novo Assembly and Annotation of the Colorado Potato Beetle, Leptinotarsa decemlineata, Transcriptome. PLoS ONE 9: e86012–e86012. 10.1371/journal.pone.0086012 24465841 PMC3900453

[16] Vidotto M, Grapputo A, Boscari E, Barbisan F, Coppe A, et al (2013) Transcriptome sequencing and de novo annotation of the critically endangered Adriatic sturgeon. BMC genomics 14: 407–407. 10.1186/1471-2164-14-407 23773438 PMC3691660

[17] Wiese J, Ohlendorf B, Blumel M, Schmaljohann R, Imhoff JF (2011) Phylogenetic identification of fungi isolated from the marine sponge Tethya aurantium and identification of their secondary metabolites. Mar Drugs 9: 561–585. 10.3390/md9040561 21731550 PMC3124973

[18] Brakhage AA (2013) Regulation of fungal secondary metabolism. Nature reviews Microbiology 11: 21–32. 10.1038/nrmicro2916 23178386

[19] Levasseur A, Lomascolo A, Chabrol O, Ruiz-Duenas FJ, Boukhris-Uzan E, et al (2014) The genome of the white-rot fungus Pycnoporus cinnabarinus: a basidiomycete model with a versatile arsenal for lignocellulosic biomass breakdown. BMC Genomics 15: 486 10.1186/1471-2164-15-486 24942338 PMC4101180

[20] Cox R, Mirkin SM (1997) Characteristic enrichment of DNA repeats in different genomes. Proc Natl Acad Sci U S A 94: 5237–5242. 9144221 10.1073/pnas.94.10.5237 PMC24662

[21] Hancock JM (2002) Genome size and the accumulation of simple sequence repeats: implications of new data from genome sequencing projects. Genetica 115: 93–103. 12188051 10.1023/a:1016028332006

[22] Linda P, Kempken F (2015) Fungal Transposable Elements; van den Berg MA, Maruthachalam K, editors: Springer International Publishing Switzerland 79–96 p.

[23] Ohm RA, Feau N, Henrissat B, Schoch CL, Horwitz BA, et al (2012) Diverse lifestyles and strategies of plant pathogenesis encoded in the genomes of eighteen Dothideomycetes fungi. PLoS Pathog 8: e1003037 10.1371/journal.ppat.1003037 23236275 PMC3516569

[24] Martin F, Kohler A, Murat C, Balestrini R, Coutinho PM, et al (2010) Perigord black truffle genome uncovers evolutionary origins and mechanisms of symbiosis. Nature 464: 1033–1038. 10.1038/nature08867 20348908

[25] Gotz S, Garcia-Gomez JM, Terol J, Williams TD, Nagaraj SH, et al (2008) High-throughput functional annotation and data mining with the Blast2GO suite. Nucleic Acids Res 36: 3420–3435. 10.1093/nar/gkn176 18445632 PMC2425479

[26] Cuenca-Estrella M, Gomez-Lopez A, Mellado E, Buitrago MJ, Monzon A, et al (2003) Scopulariopsis brevicaulis, a fungal pathogen resistant to broad-spectrum antifungal agents. Antimicrob Agents Chemother 47: 2339–2341. 12821493 10.1128/AAC.47.7.2339-2341.2003 PMC161832

[27] Xu Z, Hao B (2009) CVTree update: a newly designed phylogenetic study platform using composition vectors and whole genomes. Nucleic Acids Research 37: W174–W178. 10.1093/nar/gkp278 19398429 PMC2703908

[28] Finn RD, Bateman A, Clements J, Coggill P, Eberhardt RY, et al (2014) Pfam: the protein families database. Nucleic Acids Res 42: D222–230. 10.1093/nar/gkt1223 24288371 PMC3965110

[29] Hunter S, Jones P, Mitchell A, Apweiler R, Attwood TK, et al (2012) InterPro in 2011: new developments in the family and domain prediction database. Nucleic Acids Res 40: D306–312. 10.1093/nar/gkr948 22096229 PMC3245097

[30] Pao SS, Paulsen IT, Saier MH Jr., (1998) Major facilitator superfamily. Microbiol Mol Biol Rev 62: 1–34. 9529885 10.1128/mmbr.62.1.1-34.1998 PMC98904

[31] Walmsley AR, Barrett MP, Bringaud F, Gould GW (1998) Sugar transporters from bacteria, parasites and mammals: structure-activity relationships. Trends Biochem Sci 23: 476–481. 9868370 10.1016/s0968-0004(98)01326-7

[32] Arvas M, Kivioja T, Mitchell A, Saloheimo M, Ussery D, et al (2007) Comparison of protein coding gene contents of the fungal phyla Pezizomycotina and Saccharomycotina. BMC Genomics 8: 325 17868481 10.1186/1471-2164-8-325 PMC2045113

[33] Poggeler S, Kuck U (2004) A WD40 repeat protein regulates fungal cell differentiation and can be replaced functionally by the mammalian homologue striatin. Eukaryot Cell 3: 232–240. 14871953 10.1128/EC.3.1.232-240.2004 PMC329509

[34] Harris PV, Welner D, McFarland KC, Re E, Navarro Poulsen JC, et al (2010) Stimulation of lignocellulosic biomass hydrolysis by proteins of glycoside hydrolase family 61: structure and function of a large, enigmatic family. Biochemistry 49: 3305–3316. 10.1021/bi100009p 20230050

[35] Phillips CM, Beeson WT, Cate JH, Marletta MA (2011) Cellobiose dehydrogenase and a copper-dependent polysaccharide monooxygenase potentiate cellulose degradation by Neurospora crassa. ACS Chem Biol 6: 1399–1406. 10.1021/cb200351y 22004347

[36] Bey M, Zhou S, Poidevin L, Henrissat B, Coutinho PM, et al (2013) Cello-oligosaccharide oxidation reveals differences between two lytic polysaccharide monooxygenases (family GH61) from Podospora anserina. Appl Environ Microbiol 79: 488–496. 10.1128/AEM.02942-12 23124232 PMC3553762

[37] Hemsworth GR, Henrissat B, Davies GJ, Walton PH (2014) Discovery and characterization of a new family of lytic polysaccharide monooxygenases. Nat Chem Biol 10: 122–126. 10.1038/nchembio.1417 24362702 PMC4274766

[38] Vu VV, Beeson WT, Span EA, Farquhar ER, Marletta MA (2014) A family of starch-active polysaccharide monooxygenases. Proc Natl Acad Sci U S A 111: 13822–13827. 10.1073/pnas.1408090111 25201969 PMC4183312

[39] Lo Leggio L, Simmons TJ, Poulsen JC, Frandsen KE, Hemsworth GR, et al (2015) Structure and boosting activity of a starch-degrading lytic polysaccharide monooxygenase. Nat Commun 6: 5961 10.1038/ncomms6961 25608804 PMC4338556

[40] Saier MH Jr., Tran CV, Barabote RD (2006) TCDB: the Transporter Classification Database for membrane transport protein analyses and information. Nucleic Acids Res 34: D181–186. 16381841 10.1093/nar/gkj001 PMC1334385

[41] Schippers KJ, Sipkema D, Osinga R, Smidt H, Pomponi SA, et al (2012) Cultivation of sponges, sponge cells and symbionts: achievements and future prospects. Adv Mar Biol 62: 273–337. 10.1016/B978-0-12-394283-8.00006-0 22664125

[42] Altschul SF, Madden TL, Schaffer AA, Zhang J, Zhang Z, et al (1997) Gapped BLAST and PSI-BLAST: a new generation of protein database search programs. Nucleic Acids Res 25: 3389–3402. 9254694 10.1093/nar/25.17.3389 PMC146917

[43] Bayry J, Aimanianda V, Guijarro JI, Sunde M, Latge JP (2012) Hydrophobins—unique fungal proteins. PLoS Pathog 8: e1002700 10.1371/journal.ppat.1002700 22693445 PMC3364958

[44] Plett JM, Gibon J, Kohler A, Duffy K, Hoegger PJ, et al (2012) Phylogenetic, genomic organization and expression analysis of hydrophobin genes in the ectomycorrhizal basidiomycete Laccaria bicolor. Fungal Genet Biol 49: 199–209. 10.1016/j.fgb.2012.01.002 22293303

[45] Dubey MK, Jensen DF, Karlsson M (2014) Hydrophobins are required for conidial hydrophobicity and plant root colonization in the fungal biocontrol agent Clonostachys rosea. BMC Microbiol 14: 18 10.1186/1471-2180-14-18 24483277 PMC3922079

[46] Kis-Papo T, Weig AR, Riley R, Persoh D, Salamov A, et al (2014) Genomic adaptations of the halophilic Dead Sea filamentous fungus Eurotium rubrum. Nat Commun 5: 3745 10.1038/ncomms4745 24811710

[47] Keller NP, Turner G, Bennett JW (2005) Fungal secondary metabolism—from biochemistry to genomics. Nat Rev Microbiol 3: 937–947. 16322742 10.1038/nrmicro1286

[48] Blin K, Medema MH, Kazempour D, Fischbach MA, Breitling R, et al (2013) antiSMASH 2.0—a versatile platform for genome mining of secondary metabolite producers. Nucleic Acids Res 41: W204–212. 10.1093/nar/gkt449 23737449 PMC3692088

[49] Kramer A, Paun L, Imhoff JF, Kempken F, Labes A (2014) Development and validation of a fast and optimized screening method for enhanced production of secondary metabolites using the marine Scopulariopsis brevicaulis strain LF580 producing anti-cancer active scopularide A and B. PLoS One 9: e103320 10.1371/journal.pone.0103320 25079364 PMC4117492

[50] Tamminen A, Kramer A, Labes A, Wiebe MG (2014) Production of scopularide A in submerged culture with Scopulariopsis brevicaulis. Microb Cell Fact 13: 89 10.1186/1475-2859-13-89 24943257 PMC4075624

[51] Lukassen MB, Saei W, Sondergaard TE, Tamminen A, Kumar A, et al (2015) Identification of the Scopularide Biosynthetic Gene Cluster in Scopulariopsis brevicaulis. Mar Drugs 13: 4331–4343. 10.3390/md13074331 26184239 PMC4515620

[52] Yu ZG, Lang G, Kajahn I, Schmaljohann R, Imhoff JF (2008) Scopularides A and B, cyclodepsipeptides from a marine sponge-derived fungus, *Scopulariopsis brevicaulis* . Journal of Natural Products 71: 1052–1054. 10.1021/np070580e 18412398

[53] Kuck U, Bloemendal S, Teichert I (2014) Putting fungi to work: harvesting a cornucopia of drugs, toxins, and antibiotics. PLoS Pathog 10: e1003950 10.1371/journal.ppat.1003950 24626260 PMC3953401

[54] Tobiasen C, Aahman J, Ravnholt KS, Bjerrum MJ, Grell MN, et al (2007) Nonribosomal peptide synthetase (NPS) genes in *Fusarium graminearum*, *F*. *culmorum* and *F*. *pseudograminearium* and identification of NPS2 as the producer of ferricrocin. Current Genetics 51: 43–58. 17043871 10.1007/s00294-006-0103-0

[55] Sørensen JL, Knudsen M, Hansen FT, Olesen C, Fuertes PR, et al (2014) Fungal NRPS-dependent siderophores: from function to prediction In: Martín J-F, Garcia-Estrada C, Zeilinger S, editors. Biosynthesis and Molecular Genetics of Fungal Secondary Metabolites: Springer.

[56] Sørensen JL, Sondergaard TE, Covarelli L, Fuertes PR, Hansen FT, et al (2014) Identification of the biosynthetic gene clusters for the lipopeptides fusaristatin A and W493 B in *Fusarium graminearum* and *F*. *pseudograminearum* . Journal of Natural Products 77: 2619–2615. 10.1021/np500436r 25412204

[57] Chiang YM, Szewczyk E, Nayak T, Davidson AD, Sanchez JF, et al (2008) Molecular genetic mining of the *Aspergillus* secondary metabolome: Discovery of the emericellamide biosynthetic pathway. Chemistry & Biology 15: 527–532. 18559263 10.1016/j.chembiol.2008.05.010 PMC2494592

[58] Duressa D, Anchieta A, Chen D, Klimes A, Garcia-Pedrajas MD, et al (2013) RNA-seq analyses of gene expression in the microsclerotia of Verticillium dahliae. BMC Genomics 14: 607 10.1186/1471-2164-14-607 24015849 PMC3852263

[59] Kramer A, Beck HC, Kumar A, Kristensen LP, Imhoff JF, et al (2015) Proteomic analysis of anti-cancerous scopularide production by a marine Microascus brevicaulis strain and its UV-mutant. PLoS one (under revision). 10.1371/journal.pone.0140047 PMC4603891 26460745

[60] Fraser JA, Heitman J (2004) Evolution of fungal sex chromosomes. Mol Microbiol 51: 299–306. 14756773 10.1046/j.1365-2958.2003.03874.x

[61] Debuchy R, Turgeon BG (2006) Mating-type structure, evolution, and function in euascomycetes In: Kües U, Fischer R, editors. Growth, differentiation and sexuality. Berlin, Heidelberg: Springer pp. 293–323.

[62] Dyer PS (2007) Sexual reproduction and significance of *MAT* in the aspergilli In: Heitman J, Kronstad JW, Taylor JW, Casselton LA, editors. Sex in fungi. Washington, D.C.: ASM Press pp. 123–142.

[63] Galagan JE, Calvo SE, Cuomo C, Ma LJ, Wortman JR, et al (2005) Sequencing of *Aspergillus nidulans* and comparative analysis with *A*. *fumigatus* and *A*. *oryzae* . Nature 438: 1105–1115. 16372000 10.1038/nature04341

[64] Rydholm C, Dyer PS, Lutzoni F (2007) DNA sequence characterization and molecular evolution of MAT1 and MAT2 mating-type loci of the self-compatible ascomycete mold Neosartorya fischeri. Eukaryot Cell 6: 868–874. 17384199 10.1128/EC.00319-06 PMC1899244

[65] Wickerham LJ (1951) Taxonomy of yeasts: US Dept. of Agriculture.

[66] Kempken F, Kuck U (1996) restless, an active Ac-like transposon from the fungus Tolypocladium inflatum: structure, expression, and alternative RNA splicing. Mol Cell Biol 16: 6563–6572. 8887685 10.1128/mcb.16.11.6563 PMC231658

[67] Kollath-Leiss K, Bonniger C, Sardar P, Kempken F (2014) BEM46 shows eisosomal localization and association with tryptophan-derived auxin pathway in Neurospora crassa. Eukaryot Cell 13: 1051–1063. 10.1128/EC.00061-14 24928924 PMC4135797

[68] Margulies M, Egholm M, Altman WE, Attiya S, Bader JS, et al (2005) Genome sequencing in microfabricated high-density picolitre reactors. Nature 437: 376–380. 16056220 10.1038/nature03959 PMC1464427

[69] Knudsen T, Knudsen B (2013) CLC Genomics Benchwork 6. Available: http://www.clcbio.com. Accessed on 2013 Sept 20.

[70] Kanehisa M, Goto S, Furumichi M, Tanabe M, Hirakawa M (2010) KEGG for representation and analysis of molecular networks involving diseases and drugs. Nucleic Acids Res 38: D355–360. 10.1093/nar/gkp896 19880382 PMC2808910

[71] Wang H, Xu Z, Gao L, Hao B (2009) A fungal phylogeny based on 82 complete genomes using the composition vector method. BMC Evolutionary Biology 9. 10.1186/1471-2148-9-195 PMC3087519 19664262

[72] King N, Westbrook MJ, Young SL, Kuo A, Abedin M, et al (2008) The genome of the choanoflagellate Monosiga brevicollis and the origin of metazoans. Nature 451: 783–788. 10.1038/nature06617 18273011 PMC2562698

[73] Qi J, Wang B, Hao BI (2004) Whole proteome prokaryote phylogeny without sequence alignment: a K-string composition approach. J Mol Evol 58: 1–11. 14743310 10.1007/s00239-003-2493-7

[74] Paradis E, Claude J, Strimmer K (2004) APE: Analyses of Phylogenetics and Evolution in R language. Bioinformatics (Oxford, England) 20: 289–290. 10.1093/bioinformatics/btg412 14734327

[75] R Development Core Team (2013) R: A language and environment for statistical computing Vienna, Austria: R Foundation for Statistical computing.

[76] Finn RD, Clements J, Eddy SR (2011) HMMER web server: interactive sequence similarity searching. Nucleic Acids Res 39: W29–37. 10.1093/nar/gkr367 21593126 PMC3125773

[77] Enright AJ, Van Dongen S, Ouzounis CA (2002) An efficient algorithm for large-scale detection of protein families. Nucleic Acids Res 30: 1575–1584. 11917018 10.1093/nar/30.7.1575 PMC101833

[78] Apeltsin L, Morris JH, Babbitt PC, Ferrin TE (2011) Improving the quality of protein similarity network clustering algorithms using the network edge weight distribution. Bioinformatics 27: 326–333. 10.1093/bioinformatics/btq655 21118823 PMC3031030

[79] Veeramachaneni V, Makalowski W (2004) Visualizing sequence similarity of protein families. Genome Res 14: 1160–1169. 15140831 10.1101/gr.2079204 PMC419794

[80] Lombard V, Golaconda Ramulu H, Drula E, Coutinho PM, Henrissat B (2014) The carbohydrate-active enzymes database (CAZy) in 2013. Nucleic Acids Res 42: D490–495. 10.1093/nar/gkt1178 24270786 PMC3965031

[81] Levasseur A, Drula E, Lombard V, Coutinho PM, Henrissat B (2013) Expansion of the enzymatic repertoire of the CAZy database to integrate auxiliary redox enzymes. Biotechnol Biofuels 6: 41 10.1186/1754-6834-6-41 23514094 PMC3620520

[82] Khaldi N, Seifuddin FT, Turner G, Haft D, Nierman WC, et al (2010) SMURF: Genomic mapping of fungal secondary metabolite clusters. Fungal Genet Biol 47: 736–741. 10.1016/j.fgb.2010.06.003 20554054 PMC2916752

[83] Hansen FT, Gardiner DM, Lysøe E, Fuertes PR, Tudzynski B, et al (2014) An update to polyketide synthase and nonribosomal synthetase genes and nomenclature in *Fusarium* . Fungal Genetics and Biology in press. 10.1016/j.fgb.2014.12.004 25543026

[84] Marchler-Bauer A, Lu S, Anderson JB, Chitsaz F, Derbyshire MK, et al (2011) CDD: a Conserved Domain Database for the functional annotation of proteins. Nucleic Acids Research 39: D225–D229. 10.1093/nar/gkq1189 21109532 PMC3013737

[85] Bachmann BO, Ravel J (2009) Methods for in silico prediction of microbial polyketide and nonribosomal peptide biosynthetic pathways from DNA sequence data In: Hopwood DA, editor. Complex enzymes in microbial natural product biosynthesis Methods in enzymology. San Diego, CA, USA: Elsevier academic press inc. 10.1016/S0076-6879(09)04808-3 19374984

