# Supplementary figures and images for: De Novo Assembly and Genome Analyses of the Marine-Derived Scopulariopsis brevicaulis Strain LF580 Unravels Life-Style Traits and Anticancerous Scopularide Biosynthetic Gene Cluster
Source: PLoS One. 2015 Oct 27;10(10):e0140398. doi: 10.1371/journal.pone.0140398 (PMC4624724; doi:10.1371/journal.pone.0140398)

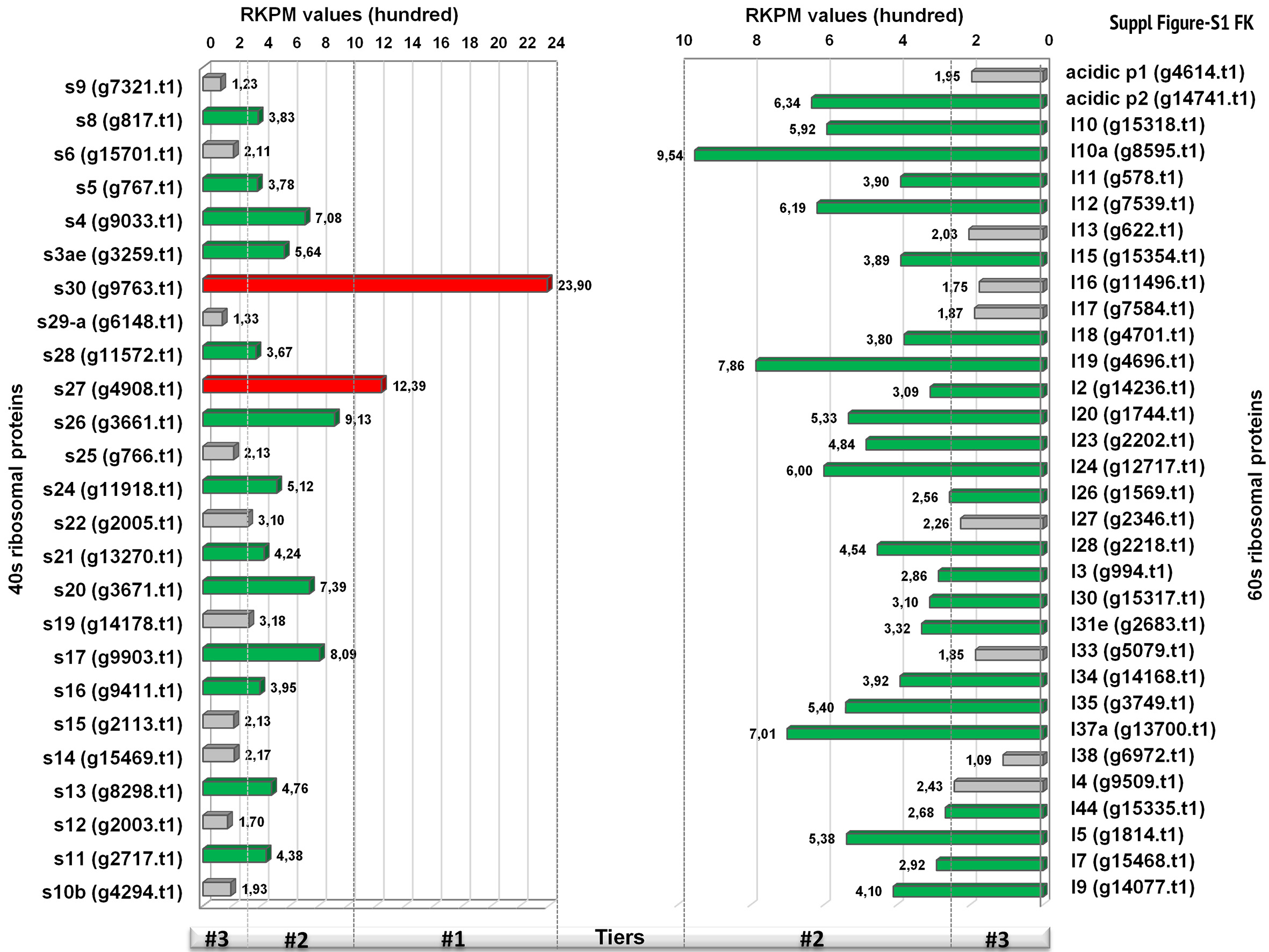

Supplement: S1 Fig — (JPG) [file pone.0140398.s001.jpg]
