# Supplementary material for: De Novo Assembly and Genome Analyses of the Marine-Derived Scopulariopsis brevicaulis Strain LF580 Unravels Life-Style Traits and Anticancerous Scopularide Biosynthetic Gene Cluster
Source: PLoS One. 2015 Oct 27;10(10):e0140398. doi: 10.1371/journal.pone.0140398 (PMC4624724; doi:10.1371/journal.pone.0140398)

Figure S2

A. Cluster1

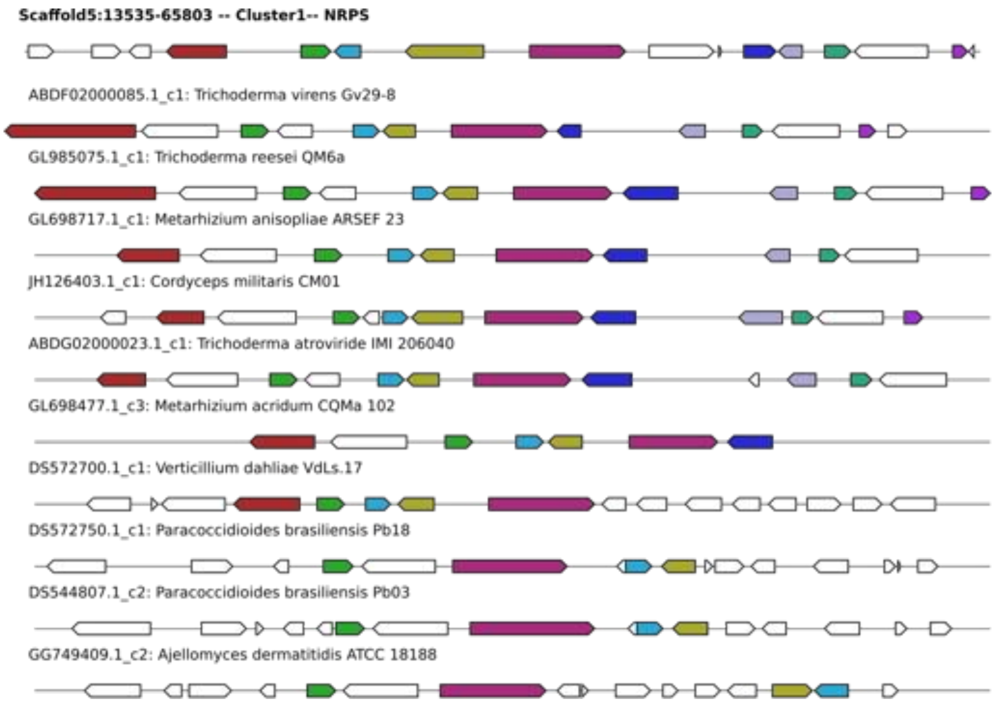

B. Cluster1

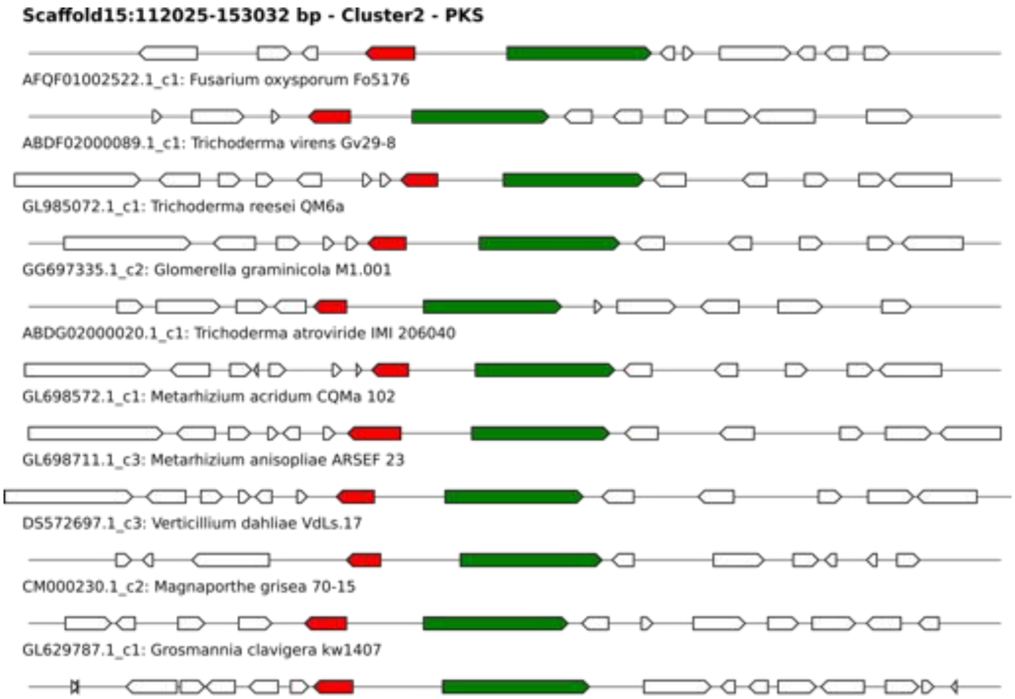

C. Cluster3

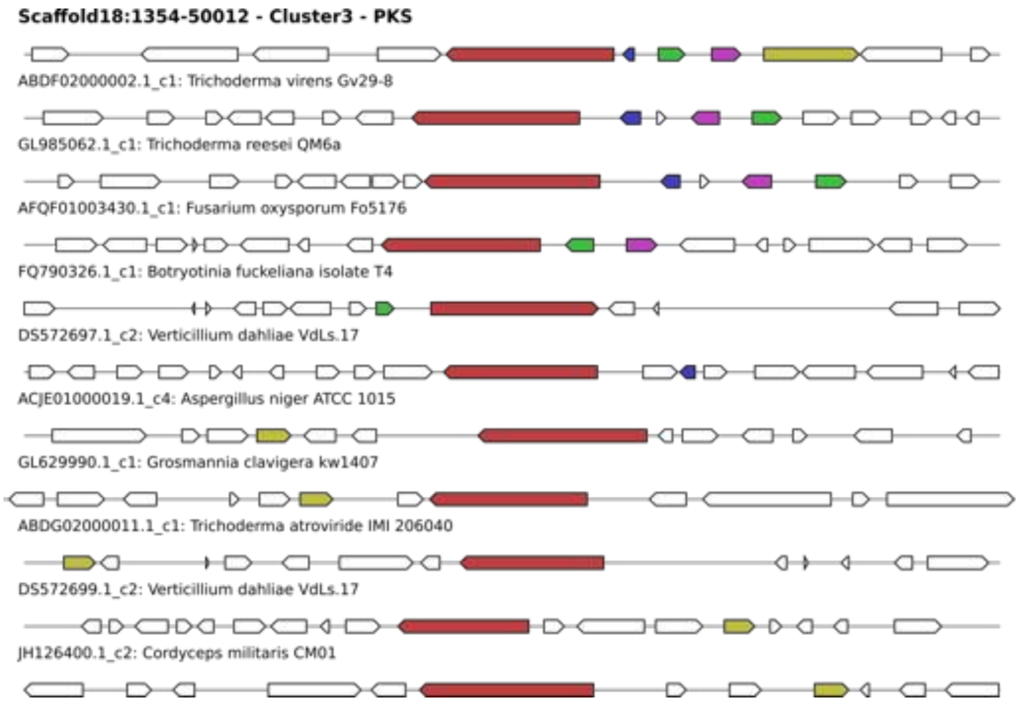

D.Cluster4

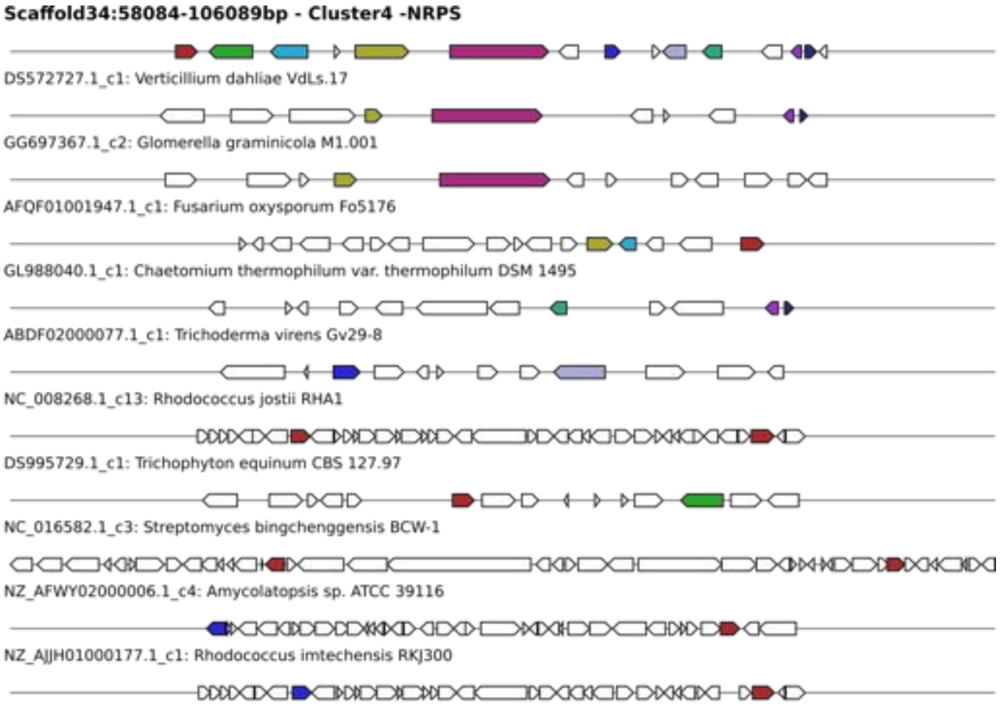

E. Cluster5

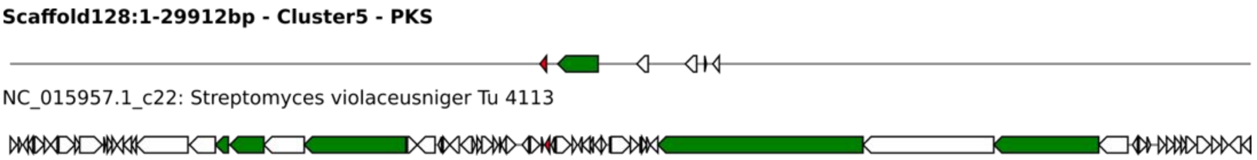

F. Cluster5

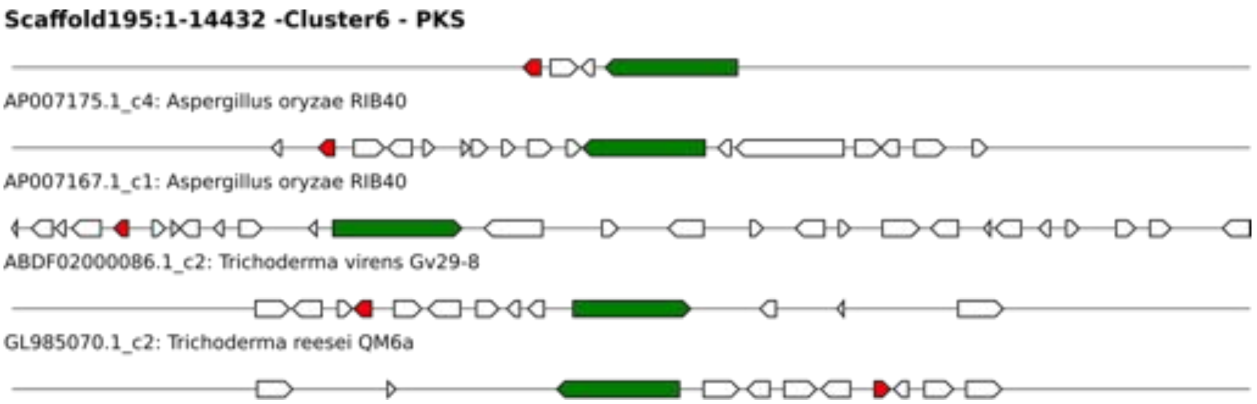

G. Cluster7

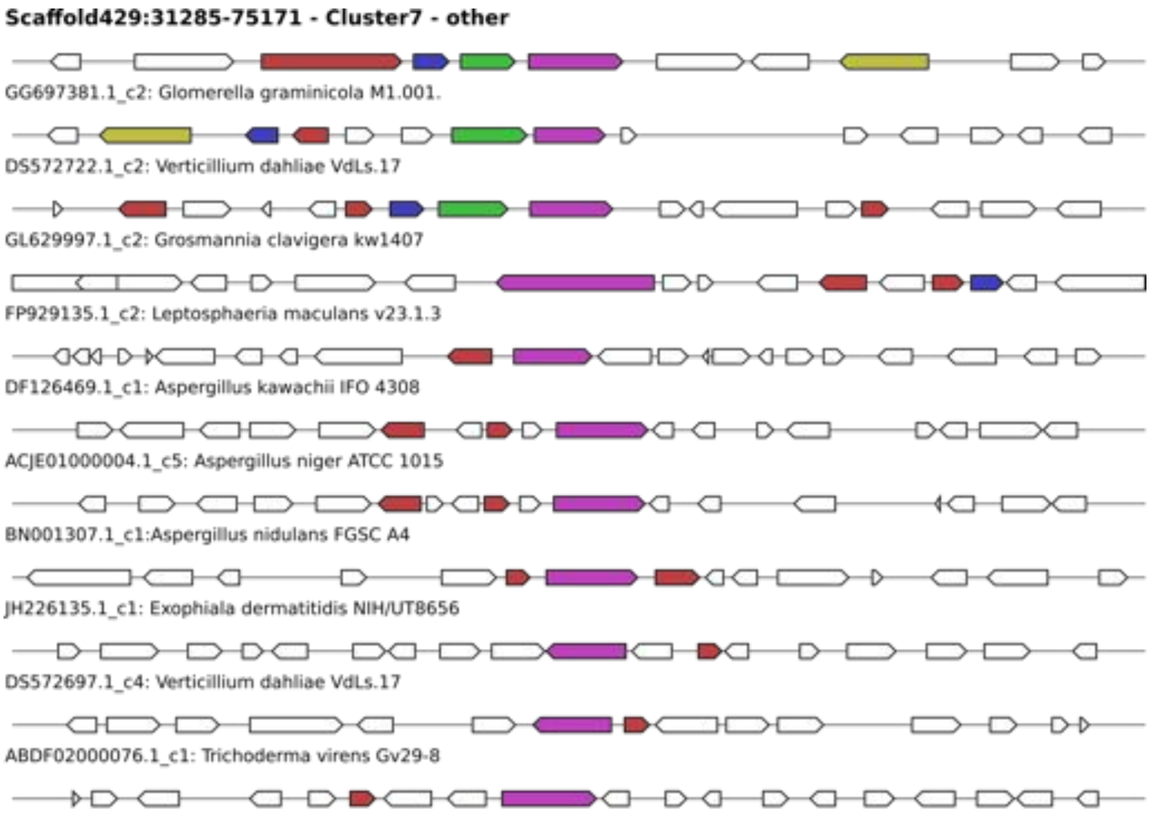

H. Cluster8

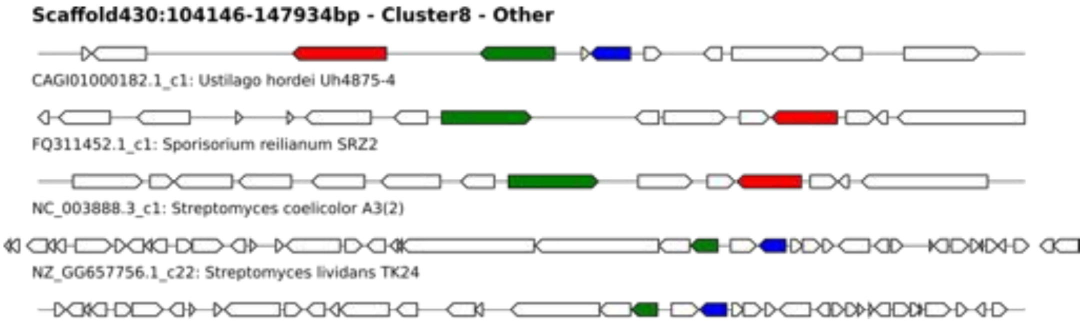

I. Cluster9

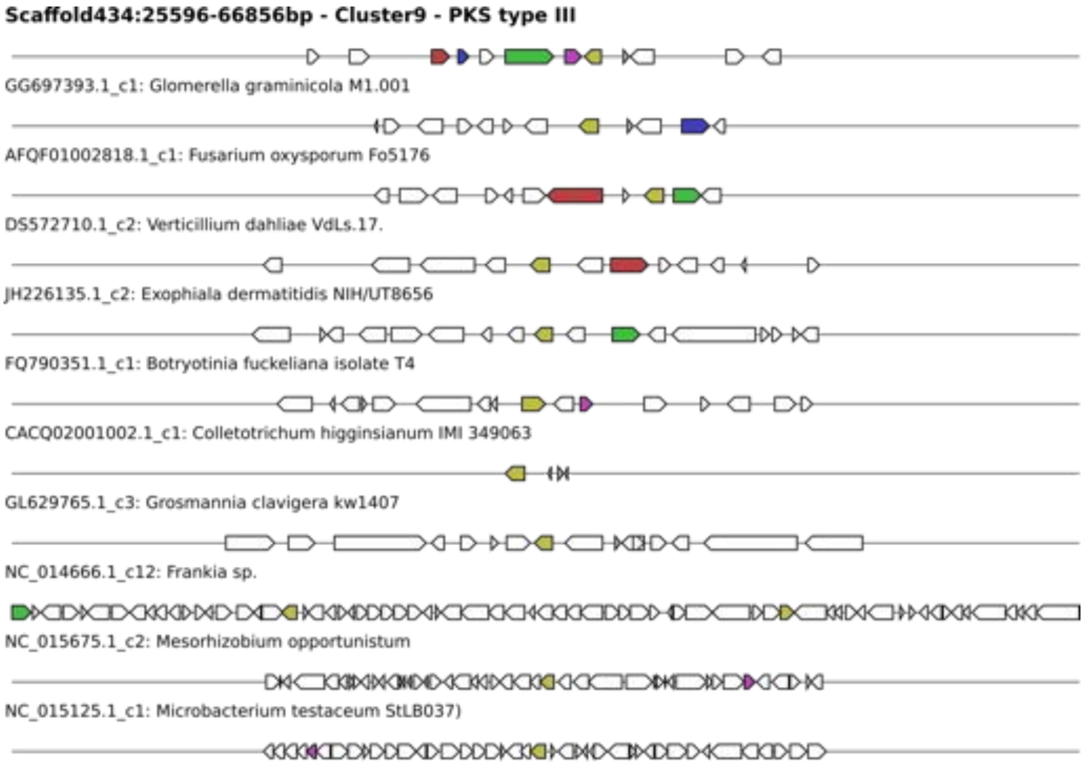

J. Cluster10

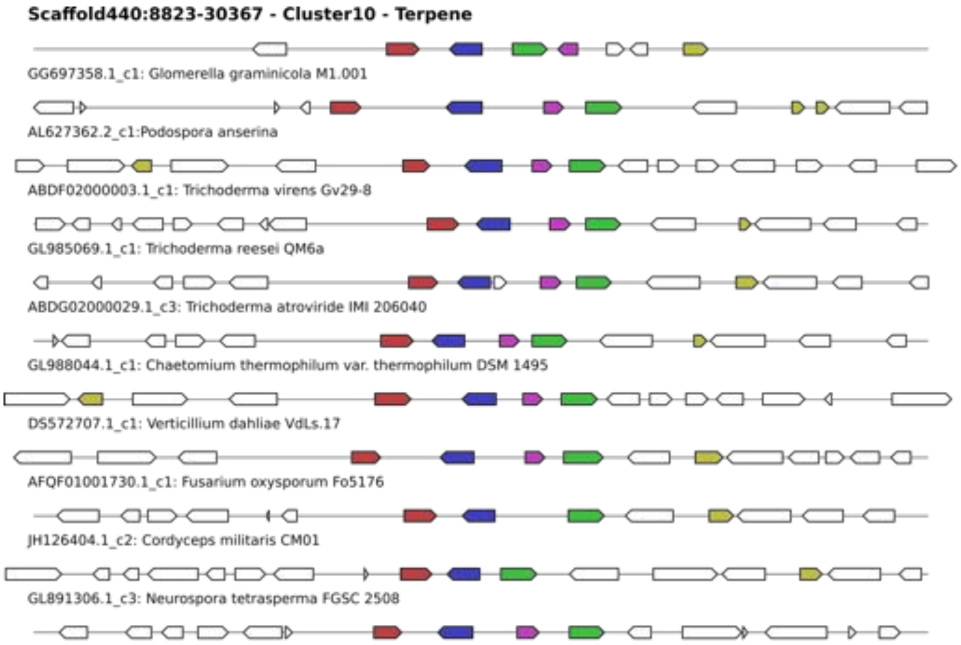

K. Cluster 11

Scaffold446 has cluster 11, which is putative terpene producing in the region 145707-166886bp

But no homologs were detected in known genome

L. Cluster12

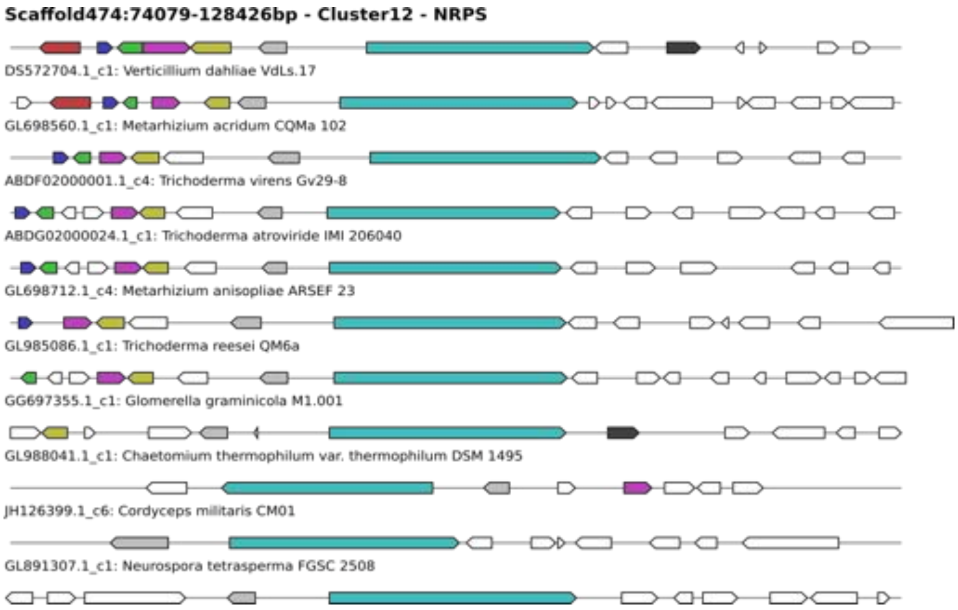

M. Cluster13

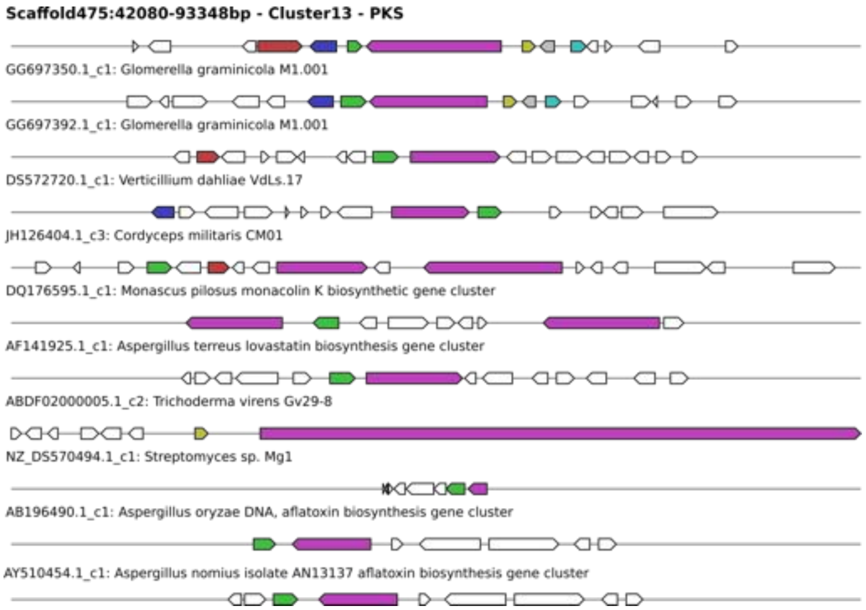

N. Cluster14

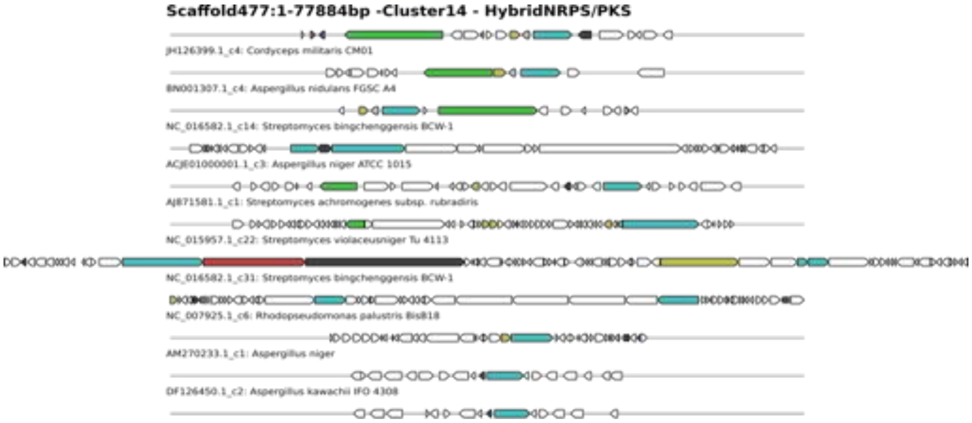

O. Cluster15

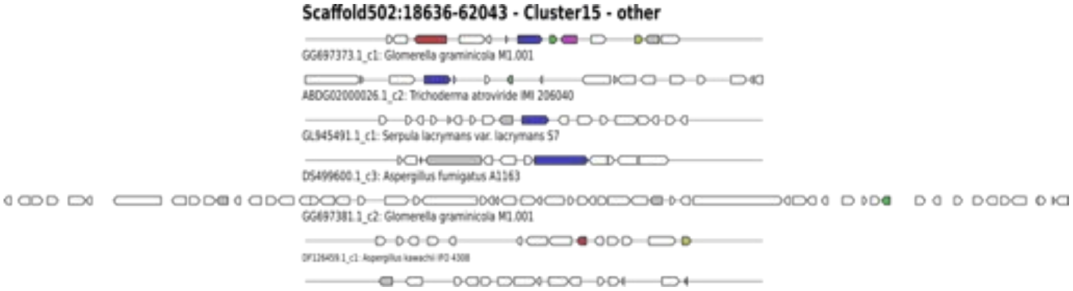

P. Cluster16

Cluster16 is localized on the scaffold553 in the region 1- 16693 bp but has no homologous clusters known in currently sequenced genomes.

Q.Cluster 17

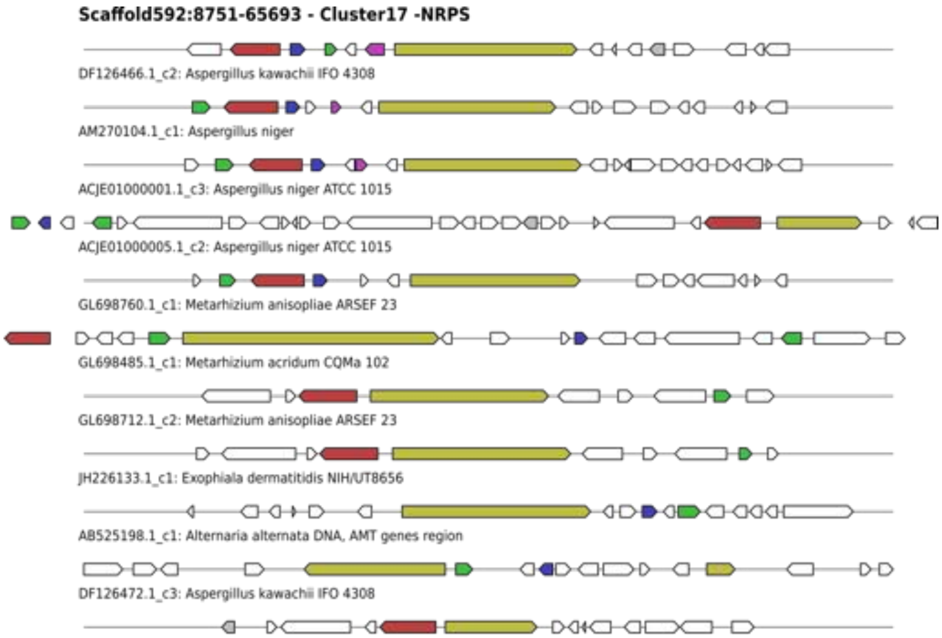

R. Cluster18

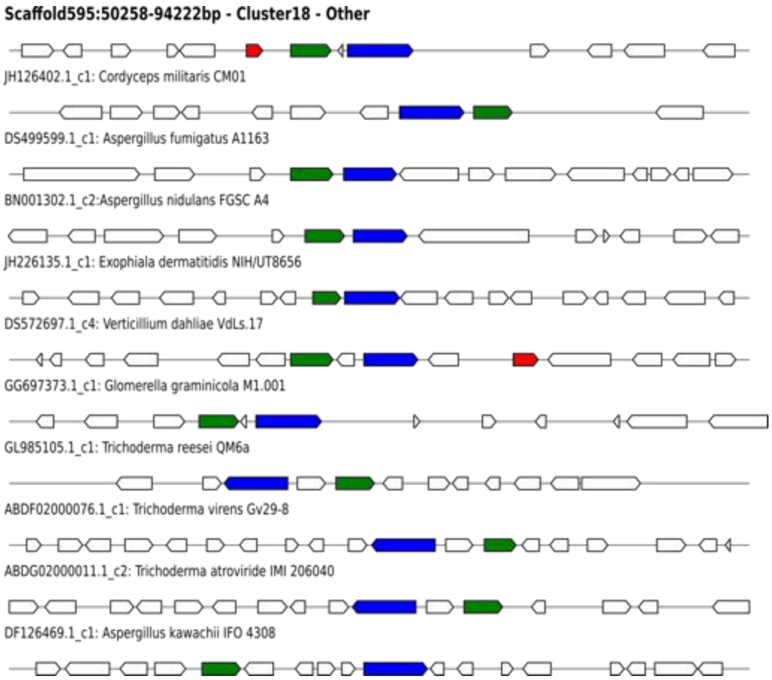

Supplement: S2 Fig — Two clusters namely cluster 11 and 16 have no homologs in known fungal genomes. (PDF) [file pone.0140398.s002.pdf]
