# Supplementary material for: De Novo Assembly and Genome Analyses of the Marine-Derived Scopulariopsis brevicaulis Strain LF580 Unravels Life-Style Traits and Anticancerous Scopularide Biosynthetic Gene Cluster
Source: PLoS One. 2015 Oct 27;10(10):e0140398. doi: 10.1371/journal.pone.0140398 (PMC4624724; doi:10.1371/journal.pone.0140398)

# Figure S8. Summary of Roche 454 reads

## 1) Overview of reads

| Region | Read count | Total bases | Average read length |
|---|---|---|---|
| 1 | 726,314 | 312,431,328 | 430.161 |
| 2 | 732,681 | 318,607,494 | 434.852 |

## 2) Read length Distribution

### A. Region/Lane 1

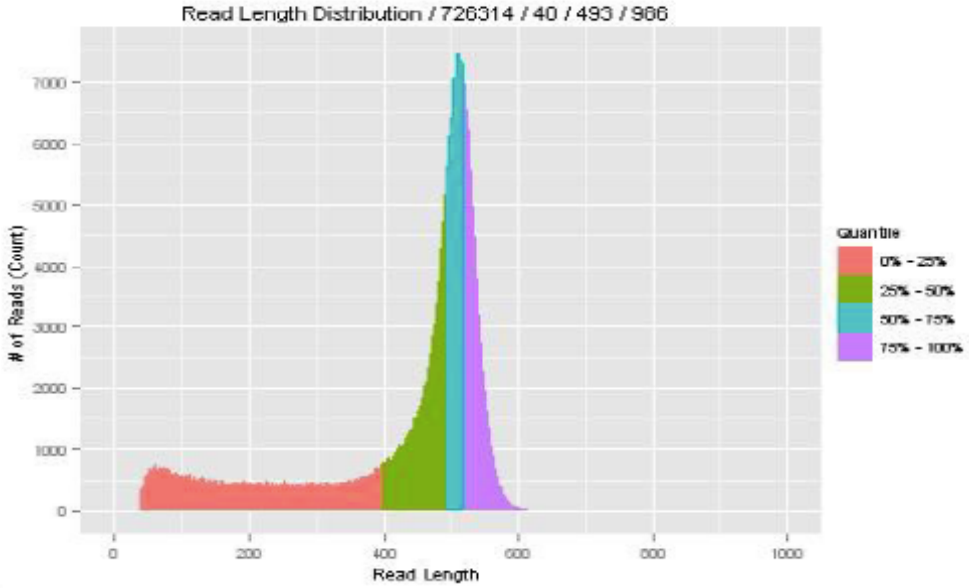


### B. Region/Lane 2

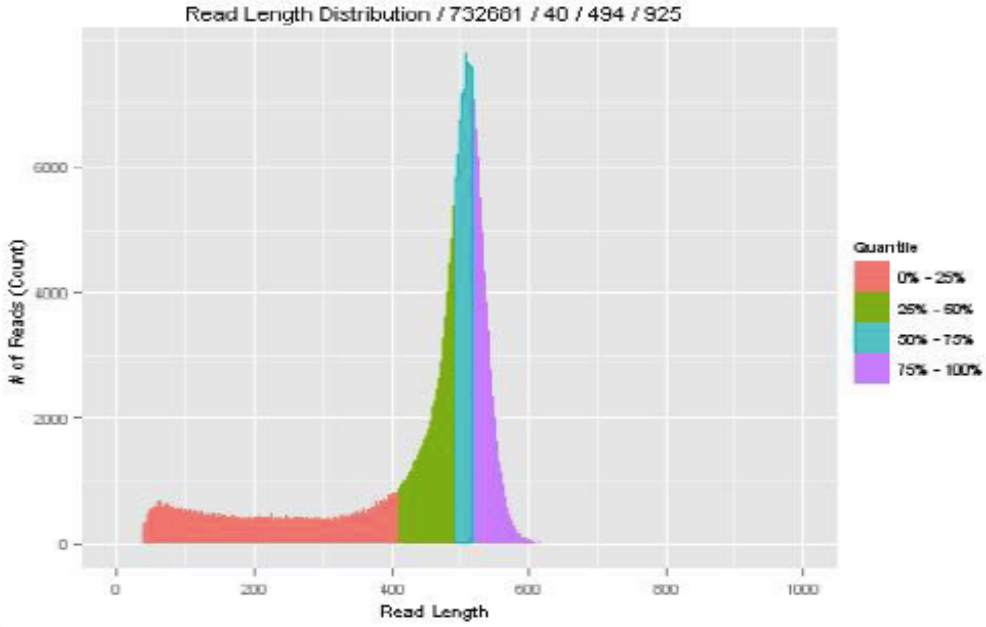

Supplement: S3 Fig — (PDF) [file pone.0140398.s003.pdf]
